# Chiral Recognition by Supramolecular Porphyrin–Hemicucurbit[8]uril-Functionalized Gravimetric Sensors

**DOI:** 10.1021/acsami.3c05177

**Published:** 2023-06-16

**Authors:** Gabriele Magna, Marko Šakarašvili, Manuela Stefanelli, Gabriele Giancane, Simona Bettini, Ludovico Valli, Lukas Ustrnul, Victor Borovkov, Riina Aav, Donato Monti, Corrado Di Natale, Roberto Paolesse

**Affiliations:** † Department of Chemical Science and Technologies, University of Rome Tor Vergata, Via Della Ricerca Scientifica 1, 00133 Rome, Italy; ‡ Department of Chemistry and Biotechnology, School of Science, Tallinn University of Technology, Akadeemia tee 15, SCI-421A, 12618 Tallinn, Harju Maakon, Estonia; § Department of Cultural Heritage, University of Salento, Via D. Birago, 48, I-73100 Lecce, Italy; ∥ Department of Biological and Environmental Sciences and Technologies, DISTEBA, University of Salento, Via per Arnesano, I-73100 Lecce, Italy; ⊥ Department of Chemistry, Sapienza University of Rome, Piazzale Aldo Moro 5, I-00185 Rome, Italy; # Department of Electronic Engineering, University of Rome Tor Vergata, Via del Politecnico 1, 00133 Rome, Italy

**Keywords:** porphyrin, hemicucurbituril, chiral recognition, quartz crystal microbalances, chemical sensors

## Abstract

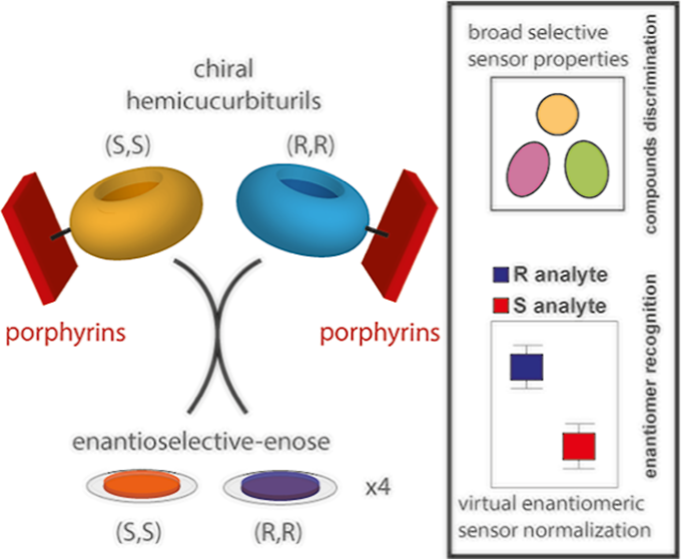

Enantiorecognition of a chiral analyte usually requires the ability to respond with high specificity to one of the two enantiomers of a chiral compound. However, in most cases, chiral sensors have chemical sensitivity toward both enantiomers, showing differences only in the intensity of responses. Furthermore, specific chiral receptors are obtained with high synthetic efforts and have limited structural versatility. These facts hinder the implementation of chiral sensors in many potential applications. Here, we utilize the presence of both enantiomers of each receptor to introduce a novel normalization that allows the enantio-recognition of compounds even when single sensors are not specific for one enantiomer of a target analyte. For this purpose, a novel protocol that permits the fabrication of a large set of enantiomeric receptor pairs with low synthetic efforts by combining metalloporphyrins with (*R*,*R*)- and (*S*,*S*)-cyclohexanohemicucurbit[8]uril is developed. The potentialities of this approach are investigated by an array of four pairs of enantiomeric sensors fabricated using quartz microbalances since gravimetric sensors are intrinsically non-selective toward the mechanism of interaction of analytes and receptors. Albeit the weak enantioselectivity of single sensors toward limonene and 1-phenylethylamine, the normalization allows the correct identification of these enantiomers in the vapor phase indifferent to their concentration. Remarkably, the achiral metalloporphyrin choice influences the enantioselective properties, opening the way to easily obtain a large library of chiral receptors that can be implemented in actual sensor arrays. These enantioselective electronic noses and tongues may have a potential striking impact in many medical, agrochemical, and environmental fields.

## 1 Introduction

Molecular systems endowed with chiral receptors are continuously developed, aiming to satisfy the challenging task of chiral recognition.^1−3^ Ideally, a single sensor can correctly detect a compound only when it is highly selective versus the target enantiomer, meaning that it must have negligible interactions with any other molecular species. Unfortunately, nearly all the receptors are commonly only partial or broadly selective to a target analyte; therefore, the recognition of target molecular species is unreliable since a single sensor response cannot be univocally correlated to the concentration of just one analyte. This behavior in sensors resembles the properties of olfactory sensory cells observed in mammals, where each receptor is sensitive to a group of volatile compounds rather than a single molecule. Albeit this limitation, olfaction may discriminate many thousands of odors, even for chiral species, relying on the differences between the sensitivity patterns of each receptor. The principle of combinatorial selectivity has been widely exploited in gas sensing to produce artificial olfaction systems,^4^ usually named electronic noses (enoses), which achieve chemical specificity, thanks to the high selectivity of global patterns of array responses, overcoming the problem of individual sensor selectivity and facilitating the design of the recognition unit.^5^

In this study, we explore the possibility of utilizing sensing films having weak selectivity for the two enantiomers of compounds into a sensor array to develop an enantioselective enose (e-enose). An e-enose should then include sensors with complementary selectivity and enantio-selectivity patterns to be able to recognize both a single compound from other ones and an enantiomer from its mirrored image, respectively. However, producing a set of chiral receptors with distinct sensing behaviors is difficult and requires a considerable synthetic effort because of the high number of reaction steps, the low yield, and the scarce versatility of chiral receptor structures, hindering the widespread diffusion of these receptors in sensorial platforms.

Here, we explore a novel approach to the development of reliable sensor arrays capable of both molecular discrimination and chiral recognition. The first obstacle related to the preparation of chiral receptors is faced by adopting a supramolecular approach as a facile route for the deposition of chiral films. In this way, we exploited the coordination of achiral metalloporphyrins with two enantiomers of cyclohexanohemicucurbit[8]uril {(*R*,*R*)-and (*S*,*S*)-cycHC[8]}^6,7^ as chiral effectors. Inducing chirality through supramolecular interactions has the great benefit of being less laborious from a synthetic point of view since it is possible to use forming units that are relatively easy to prepare and design according to the application.

In this context, we have combined the binding properties of metalloporphyrins, which have widely been used for the realization of molecular receptors in chemical sensors^8−27^ with the facile preparation of enantiomerically pure form of cycHC[8] as chiral macrocyclic effectors.^6,28^ Hemicucurbiturils can bind electron-rich species inside^29,30^ the cavity and electron-deficient species outside their ring.^31,32^ We exploit the latter property to promote the interaction with the metal ions coordinated to several porphyrins to realize chiral hybrid adducts in the solid state. In this way, we have designed solid-state sensors where a chiral block, cycHC, induces chirality in different metalloporphyrins, which in turn act as receptors. The variations in porphyrin structures allow the production of a set of receptors with overlapping and complementary sensitivity patterns versus different volatile organic compound (VOC) classes.

**Figure 1:**
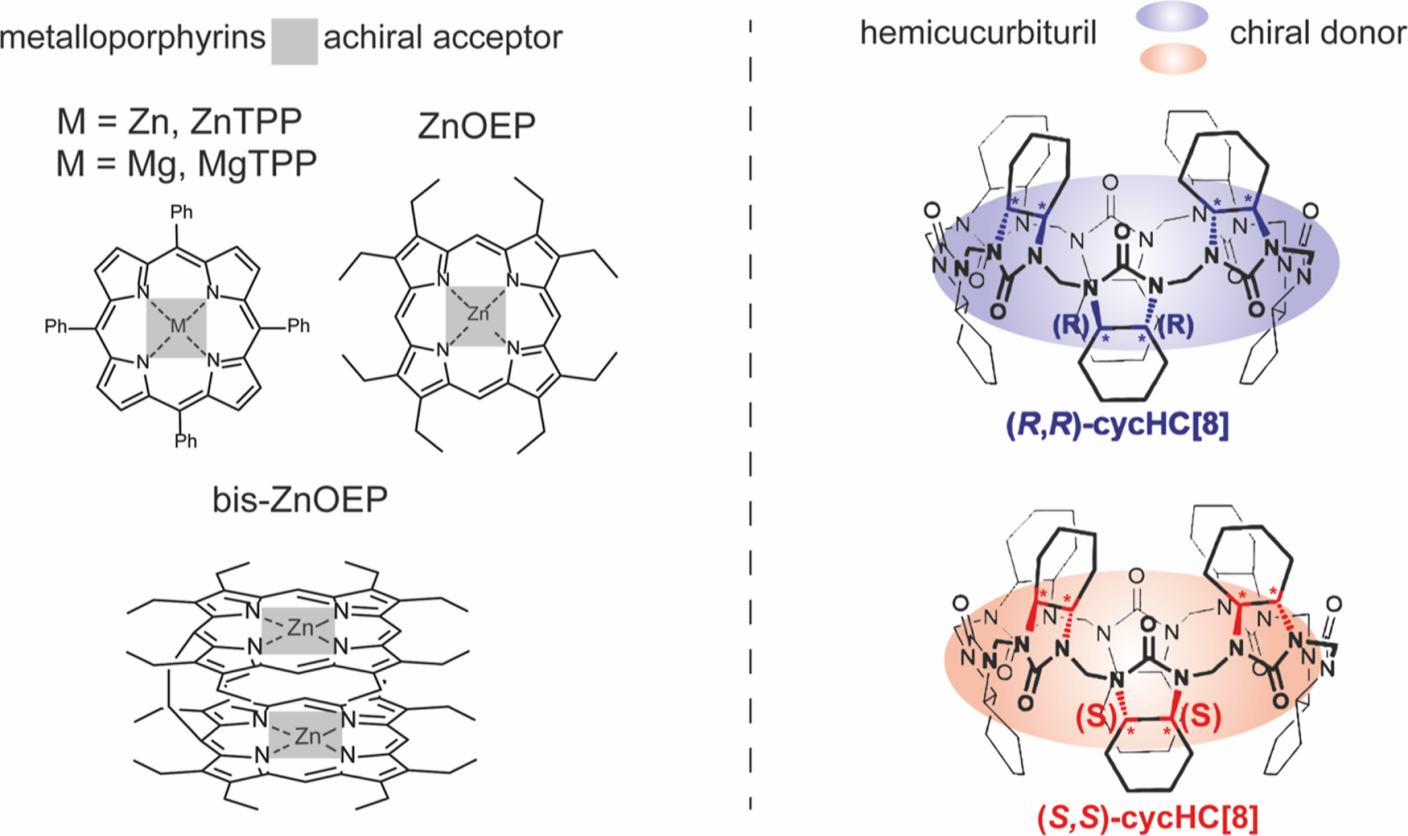
Structures of cycHC[8] enantiomers and metalloporphyrins utilized to produce chiral sensing films.

**Figure 2:**
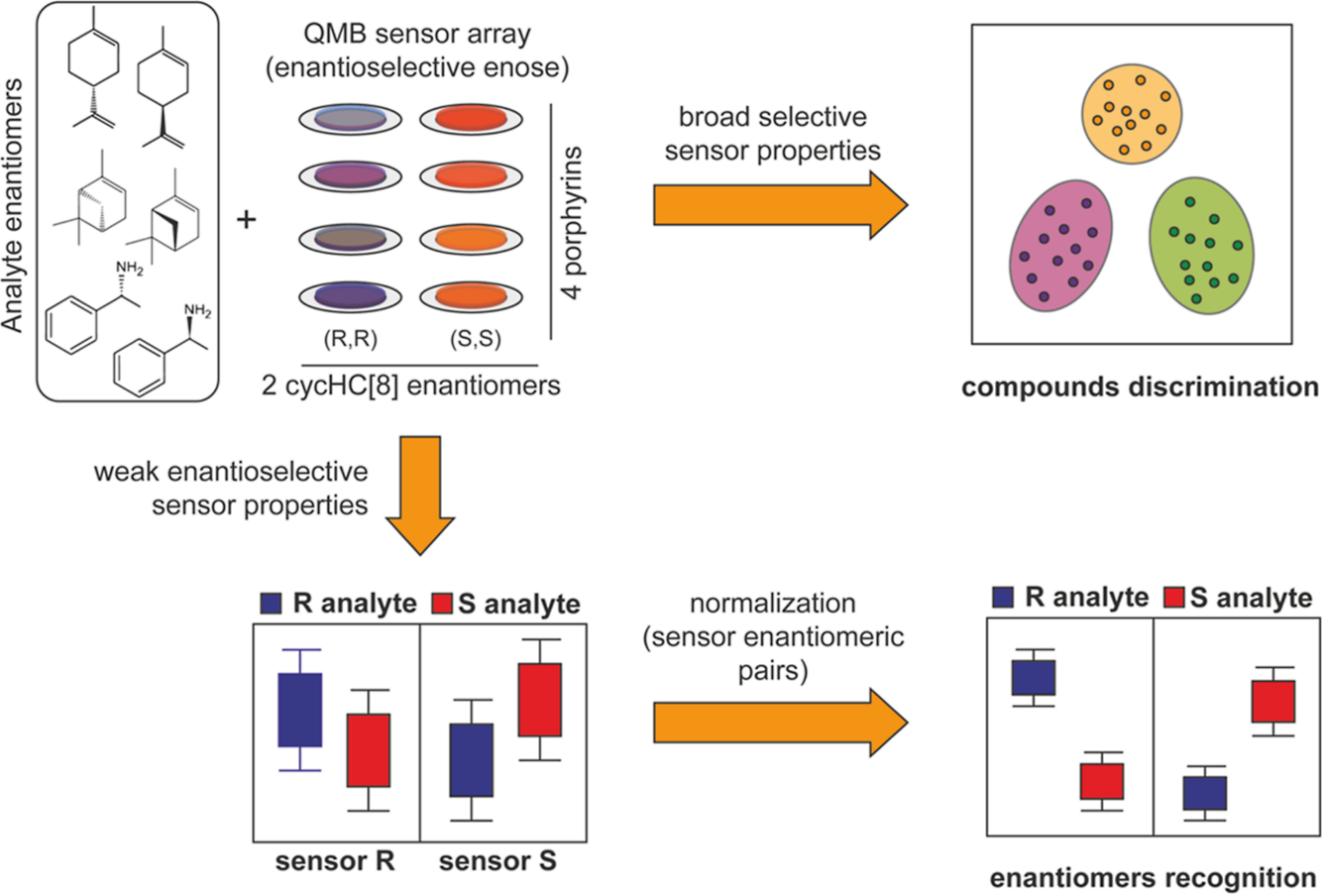
Schematic representation of the proposed enantioselective electronic nose. Different metalloporphyrins allow compound discrimination, whereas chiral induction by hemicucurbituril macrocycles permits recognition of the enantiomers after an ad hoc normalization.

At the same time, mirrored chiral effectors in adducts allow sensors to be selective toward the handedness of analytes; although the selectivity toward the enantiomeric pairs is only partial, even a small difference can be successfully exploited for enantiorecognition, hence mimicking the differential absorption of polarized light utilized in the circular dichroism spectroscopy. This approach is schematically represented in Figures 1 and 2, and it will be detailed in the following Results and Discussion section. Because the achiral porphyrin is a unit responsible for the enantiorecognition properties of films, variations on the substituents and coordinated metal ion of the porphyrin allow to easily produce a large set of chiral sensors, significantly reducing the synthetic effort, with a wide library of metalloporphyrins available.

To the best of our knowledge, this approach has not been reported in the literature yet, and it could represent a breakthrough in the realization of chiral sensor arrays. In this work, we have successfully tested this approach for the chiral recognition of enantiomeric pairs of some model analytes.

## 2 Experimental Details

### 2.1 Materials and Methods

Reagents and solvents were purchased from Sigma-Aldrich, Merck, or Carlo Erba and were used as received. Metalloporphyrins and hemicucurbit[8]uril units were prepared following literature procedures.^6,33^ (*R*)-(+)-Limonene (97% purity), (*S*)-(−)-limonene (96% purity), (+)-α-pinene (98% purity), (−)-α-pinene (98% purity), (*R*)-(+)-α-methylbenzylamine (98% purity), and (*S*)-(−)-α-methylbenzylamine (98% purity) were commercially available from Sigma-Aldrich. Solvents used for spectroscopic measurements were of spectroscopic grade. UV–vis spectra were measured with a Cary 100 spectrophotometer. CD spectra were obtained on a JASCO J-1500 spectrophotometer, equipped with a thermostated cell holder set at 298 K, and purged with ultrapure nitrogen gas. Linear dichroism contribution (LD) has been found to be <0.0004 DOD units in all of the cases examined.

### 2.2 Preparation of Solid Films on Glass for Circular Dichroism Studies

Films on glasses were obtained by drop-casting dichloromethane solutions of the adducts. For this purpose, 0.4 mM metalloporphyrins (Figure 1) were dissolved in a 0.2 mM solution of either (*R*,*R*) or (*S*,*S*)-cycHC[8] in CH_2_Cl_2_. Films on glass were obtained either by drop-casting 50 μL of solutions or by the spin coating technique by repeatedly dropping 10 μL of solution at 1500 rpm.

### 2.3 Quartz Microbalances

Quartz microbalances (QMBs) are piezogravimetric devices that may act as mass transducers since a load produces a decrease in their resonance frequency linearly correlated to mass increment in the case of small perturbations.^34^ The QMBs utilized are AT-cut quartzes oscillating at the first-harmonic frequency of approximately 20 MHz (KVG GmbH). The quartz diameter is 9.0 mm coated by 5.0 mm gold electrodes of diameter on both faces. Such quartzes have a theoretical mass sensitivity of about 7.20 Hz/ng with a minimum reliable frequency measurement of 0.1 Hz.^35^

Sensing films were deposited onto the QMB electrodes by drop-casting the solution in CH_2_Cl_2_. A 5 μL drop was cast onto the gold electrode waiting for the solvent evaporation. Casting was iterated up to reach at least 15 kHz of material for each side of QMB.

The experimental setup for sensor measurements is depicted in Figure S1. Sensors were allocated in an airtight chamber with a gas inlet and outlet. Each QMB was connected to an electronic oscillator circuit, and in-house designed electronics allowed the measurement of oscillation frequencies of up to 12 piezogravimetric elements. Sensor responses were measured by exposing the systems to different concentrations of saturated vapors of (*R*)- and (*S*)-limonene, (*R*)- and (*S*)-2-phenylethylamine, and (1*R*,5*R*)- and (1*S*,5*S*)-2-pinene enantiomers diluted in a pure nitrogen gas carrier. Saturated vapors were obtained by bubbling pure nitrogen gas in the liquid samples. At 25 °C, the concentrations of saturated vapors of limonene, 2-pinene, and 1-phenylethylamine are 1980,^36^ 5822,^37^ and 657 ppm,^38^ respectively. Flux and vapor dilutions were governed by a system of mass flow controllers (MKS). The total flux at the inlet of the sensor cell was always kept constant at 200 standard cubic centimeters per minute. The temperature of the whole system (liquid sample, gases, and sensors) was always kept at 25 °C, and ambient humidity was maintained at around 10% for the whole experiment.

The sensor array was exposed to each concentration four times, and a random order was used for each sequence replica. Each measurement consisted of a gas exposure (5 min) followed by exposure to the carrier gas (20 min). In the measurement sequence, the enantiomers are alternately delivered at the same concentration to reduce time influence in the responses. Sensor signals were totally reversible.

## 3 Results and Discussion

### 3.1 Spectroscopic Characterization

First, the metalloporphyrin·cycHC adducts have been investigated in solution phase and as solid-state films. The possibility of transferring the chiral information from enantiopure cycHC[*n*] (*n* = 6, 8) to achiral Zn-porphyrin derivatives was previously demonstrated in nonpolar solvents, exploiting the favorable interactions between the electron-deficient porphyrin Zn^2+^ ion and the electron-rich carbonyl oxygen on the outer surface of cycHCs (Zn^2+^···O=C).

In the case of ZnTPP·(*R*,*R*)-cycHC[8], crystallographic data showed a complex structure where one molecule of cycHC[8] is bound to two porphyrins, packed in a manner that each ZnTPP is separated from another porphyrin by cycHC[8]. This creates a chiral environment next to all porphyrins in the crystal.^31^

On the other hand, bis-ZnOEP has demonstrated outstanding chirality sensing properties for amines and alcohols due to the induction of characteristic electronic circular dichroism (ECD) signals.^39^ Chirality induction in bis-ZnOEP was also observed by complexation with cycHC[*n*]s.^32^ Based on stoichiometry in the crystal structures obtained in the case of ZnTPP, we combined cycHC[8] enantiomers with different metalloporphyrin derivatives (Figure 1) in a 1:2 molar ratio to produce chiral films where the planar configuration of porphyrin derivatives combined with the oxophilicity of Mg and Zn metal ions promote coordination and, consequently, chiral induction.

Chiral films were obtained via deposition of the complexes’ solution onto glass slides using drop-casting or spin coating techniques without denoting any relevant difference in optical and chiroptical properties depending on the deposition technique employed. Thus, spin-coated films have been utilized for characterization since the films appeared to be more uniform, avoiding the presence of clusters or coffee-rings phenomena due to solvent volatility.

**Figure 3:**
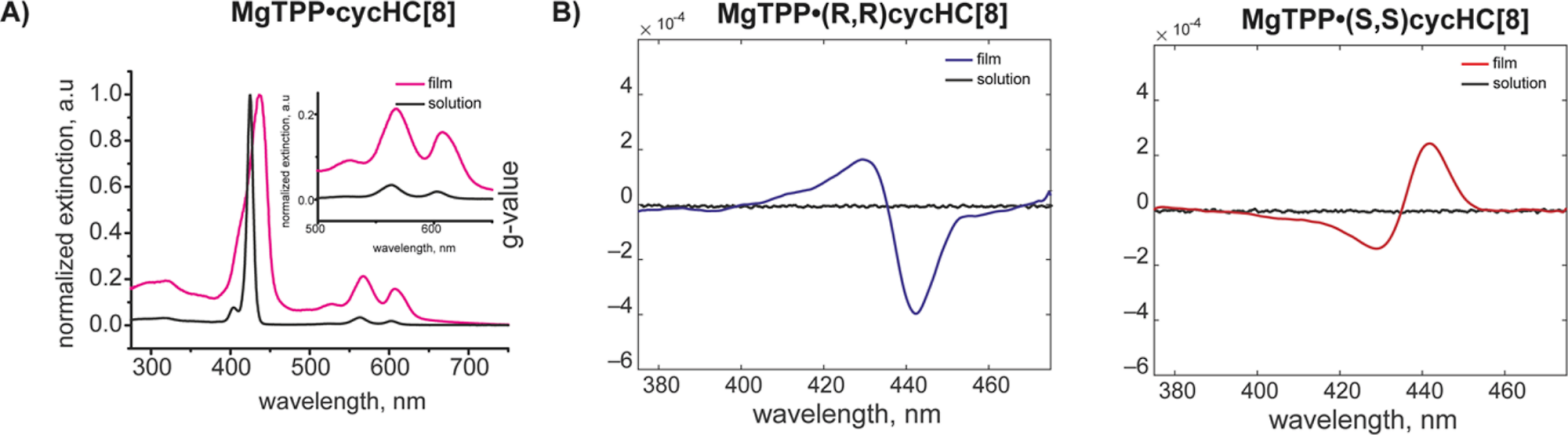
(A) UV–vis spectra of 1:2 mix of chiral cycHC[8] and MgTPP porphyrin in CH_2_Cl_2_ solution and on spin-coated glass slides. (B) ECD spectra of their enantiomeric adducts on spin-coated glass and solution.

UV–vis spectra show broadening and bathochromic shift (see Table S1) of the Soret band of all metalloporphyrins•cycHC[8] films as compared with the corresponding spectra in CH_2_Cl_2_ solution (Figure 3A). Q bands were also red-shifted, suggesting the formation of various porphyrin aggregates in the films as a natural consequence of solvent evaporation. Please note that in the case of aggregation, as in films, the B-band of porphyrins (400–450 nm) decreases in intensity much more than the Q-bands (500–650 nm). Since absorbances are normalized between 0 and 1, the lowering of the main band produces an apparent increase of other bands (as appearing in the inset in Figures 3A and S2).

Concerning the CD characterization, in CH_2_Cl_2_, the solutions of adducts utilized to prepare sensing layers do not show any ECD features in the visible range. For example, in solution, we can observe ECD signal for the complexes of metalloporphyrin and cycHC[8] with a high excess of cycHC[8], which is necessary due to the micromolar concentration limitation of porphyrins in these measurements.^31^ In our case, the porphyrin/cycHC ratio is 2:1. Conversely, in the case of solid films, this limited amount of HC is sufficient to induce chirality once the solvent evaporates. CD spectroscopy discloses the emergence of dichroic bands in all films that contain hemicucurbituril-porphyrin adducts, showing Cotton effects of the opposite sign in the case of antipodal cycHC[8] (Figures 3B and S2). Since HCs are not absorptive in the visible range, the appearance of CD signal in this range is only due to the chirality induction in metalloporphyrins. All UV–vis and ECD spectral features for the eight systems investigated, both in solution and solid-state, are summarized in Table S1. From a chemical sensor point of view, producing chiral films without a high excess of the hemicucurbituril inducers enables the possibility of simply modulating the chemical sensitivity by structural changes only in metalloporphyrins, which possess a high synthetic versatility. As previously mentioned, the possibility of effortlessly producing sensors with different sensitivity patterns is the fulcrum for recognizing different compounds or mixtures in typical enoses. Furthermore, we observed that the CD features manifest on different quartz substrates and deposition methods, proving this approach to be much more robust than films produced with chiral porphyrins, where, for example, solvent, substrate, and deposition techniques strongly influence the possibility of having supramolecular chiral films.^40^

In detail, the presence of either (*S*,*S*) or (*R*,*R*)-cycHC[8] produces the (+/−) or (−/+) bisignate ECD features in the corresponding porphyrin absorption region (Figure 3B). The ECD patterns of films made by two enantiomers may not be perfectly mirrored due to local inhomogeneities that may alter the spectral profiles. Indeed, the ECD signals in anisotropic solid-state samples (including thin films) depend not only upon the differential absorbance of circularly polarized light but also on other film local properties, such as the presence of LD or birefringence contributions. Thus, distinct ECD profiles may be obtained after the formation of films by spin coating or drop-casting due to metastable or kinetically entrapped species after solvent evaporation.^41^ Last, in the case of bis-ZnOEP adducts, the ECD pattern is more complex, likely due to the presence of anti-syn conformations of bis-porphyrins in equilibrium with each other and oppositely oriented inter- and intramolecular exciton couplings of corresponding porphyrin electronic transitions in the solid state.^33^ Also, in the UV–vis spectra of this adduct film, the broadening of the Soret band derives from the superimposition of the absorptions of both conformations, which are difficult to distinguish due to the close energy position of the corresponding electronic transitions. Perhaps, it can only be seen as some shoulders (if any).

### 3.2 Sensor Measurements

**Figure 4:**
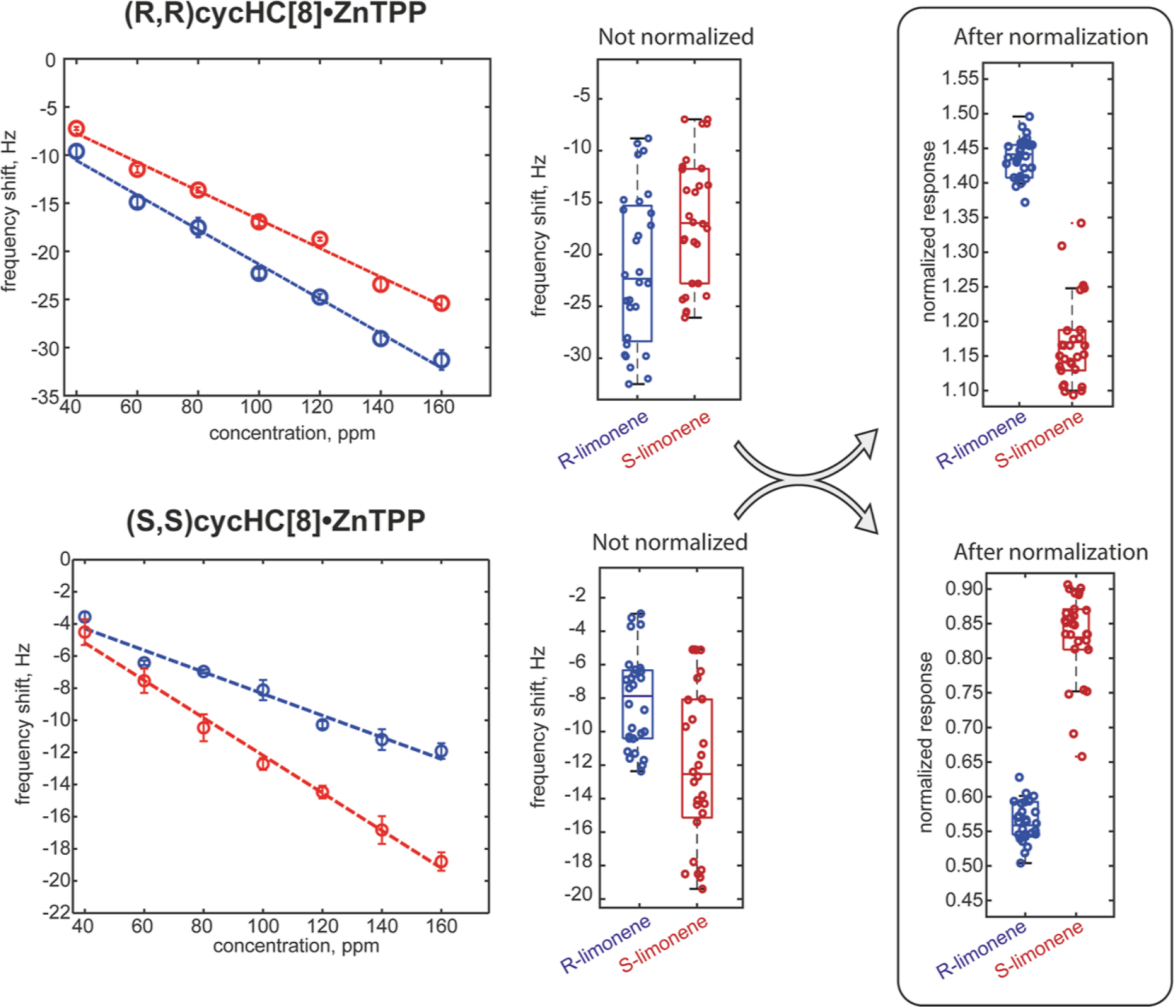
Examples of responses and data distributions of compounds tested with and without normalization involving enantiomeric pair of sensors. Responses of cycHC[8]·ZnTPP sensors toward limonene.

**Figure 5:**
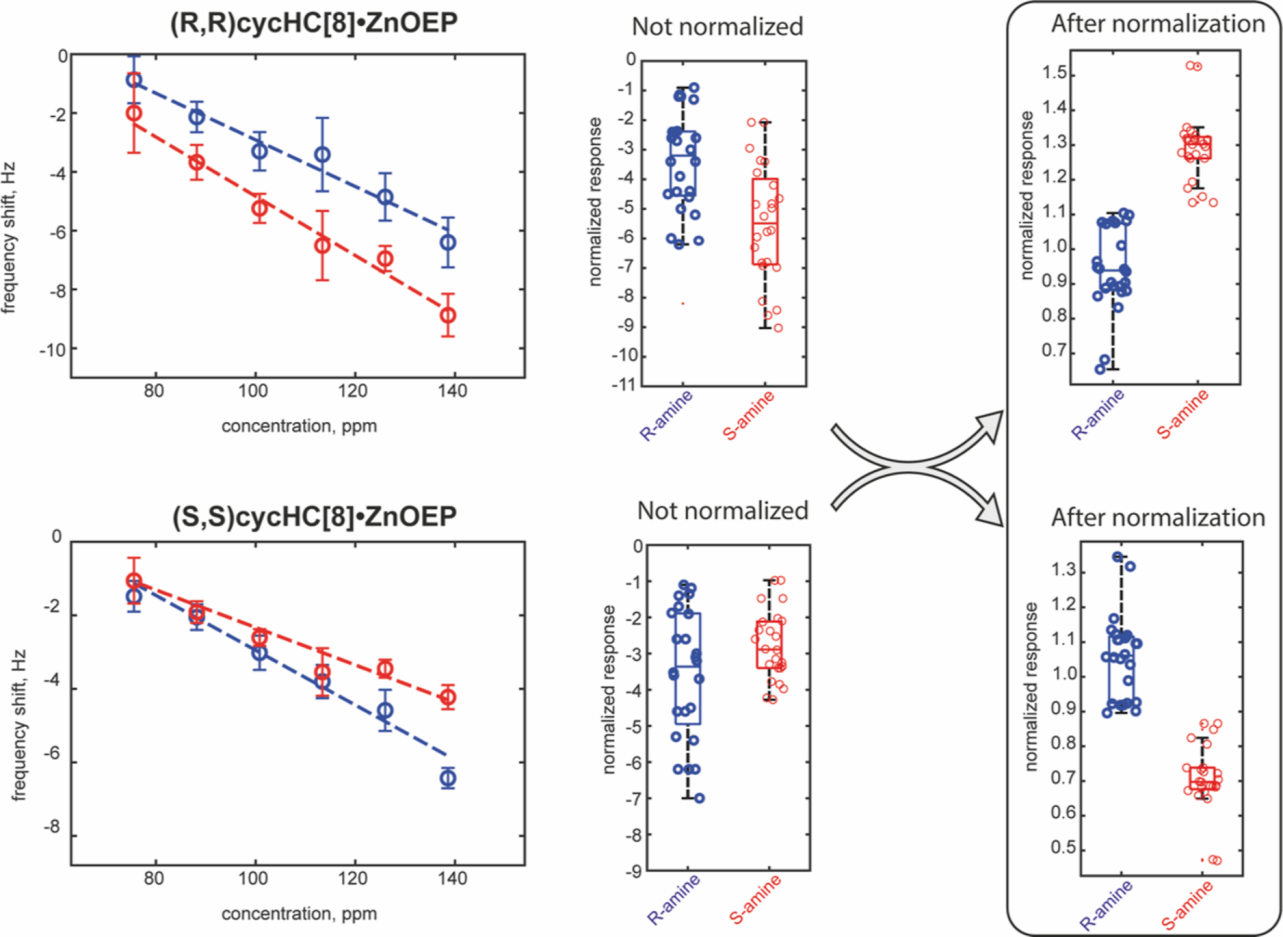
Examples of responses and data distributions of compounds tested with and without normalization involving enantiomeric pair of sensors. Responses of cycHC[8]·ZnOEP sensors toward 1-phenylethylamine.

Subsequently, the films were deposited onto QMBs, and the sensors were exposed to three pairs of enantiomerically pure vapors, as reported in Figure S1. Two achiral sensors based on ZnTPP and bis-ZnOEP films were included as references in the sensor array. VOC concentrations tested are listed in Table S2. Remarkably, use of gravimetric sensors permits estimating and dosing the sensing materials deposited onto the surface, guaranteeing a high reproducibility of the sensors. As reported below, the possibility of obtaining comparable (*R*,*R*)- and (*S*,*S*)-sensor pairs will be crucial for data normalization. Deposition details are reported in Table S3. We selected two terpenes and one chiral amine as representative examples of chiral analytes, which are commercially available in both enantiomeric forms. Limonene has been a very useful model compound in our previous tests, and thanks to its linear structure, it can easily diffuse inside the porphyrin aggregates.^18^ Conversely, 2-pinene has a reduced capacity of π-interactions due to its single double bond, and it is bulkier than limonene due to the dimethyl-methylene bridge, making the adsorption of this compound to be confined only to the superficial film sites.^42,43^ Finally, the planar aromatic 1-phenylethylamine combines π–π interactions with additional NH_2_-coordination to the metal center that may foster the absorption into the film. Figures S3–S5 report the characteristic curves of the sensors to limonene, 1-phenylethylamine, and 2-pinene, respectively. In the case of limonene, almost all chiral sensors show a significantly different response to the two enantiomers. Interestingly, all Zn-porphyrins•(*R*,*R*)-cycHC[8] films show a preference for (*R*)-limonene, whereas MgTPP•(*R*,*R*)-cycHC[8] has an opposite behavior, likely due to the difference in the coordination ability of Zn- and Mg-porphyrins. Specular effects were observed in the case of films based on the (*S*,*S*)-cycHC[8] counterpart. At the same time, as expected, achiral films, namely ZnTPP and bis-ZnOEP, do not display any statistically relevant enantiorecognition ability. Albeit the overall sensing mechanism is complicated and will be deeply investigated/modeled in future works, this outcome empirically suggests that the chiral recognition mechanism is based on the interaction of a chiral guest with porphyrins, while the role of cycHC is just to induce chirality in the macrocycle. The lack of enantioselectivity of cycHC toward tested chiral compounds enforces this hypothesis (see Supporting Information, Figures S6–S8). Moreover, pure hemicucurbituril films showed a strong water sensitivity, which notably reduced the enantioselectivity in comparison to that observed in all films produced with adducts of porphyrins–cycHC (see Supporting Information). In the case of 2-pinene (Figure S9), all sensors show a poor response without showing enantioselectivity. This outcome agrees with the previous experiments where the diffusion of analytes into the film emerged as a guiding condition for having enantioselectivity with porphyrins on QMBs.^23^ Thus, pinene enantiomers are essentially adsorbed into the superficial portion of the sensing layers, as highlighted by the low sensitivity of all films to these vapors. From the characteristic curves of 1-phenylethylamine, the differences between the responses to the (*R*,*R*)- and (*S*,*S*)-enantiomers appear to be evident only in the sensors based on ZnOEP and bis-ZnOEP, and this separation increases at higher concentrations. As an example, Figures 4 and 5 report the responses of two enantiomeric pairs of sensors based on ZnTPP and ZnOEP toward limonene and 1-phenylethylamine enantiomers. The responses vs concentrations curves evidence a separation between the responses of the two enantiomers regression curves (left plot in each panel). This means that if we consider a fixed concentration, the samples of two enantiomers produce different sensor responses. However, when changes in the sample concentration are considered, it is evident that the same value of sensor response can be produced by a higher concentration of the analyte to which the sensor is less sensitive or by a lower concentration of the analyte to which the sensor is most sensitive. Thus, the variation in sample concentration produces an overlap between the responses of the same sensor to both enantiomers. As a consequence, when we consider the distributions of sensor responses to both enantiomers of the analyte, the boxplots evidence that the mean values of distributions are different, but the dispersion of data due to the concentration makes recognizing one of the two enantiomers impossible. This is visualized in the boxplots reported in Figures 4, 5, and S10.

It is worthy of note that concentration is often ignored or overlooked in recognition tasks. However, it has a central role since a relatively wide range may considerably hinder the possibility of distinguishing two different analytes. To overcome this fundamental problem in enantiomer recognition, we propose a novel normalization protocol to assess the chiral nature of analytes, disregarding their concentration even in the case of broad chemical selectivity and low enantioselectivity.

### 3.3 Virtual Racemic Sensor Normalization

In the case of mass sensors such as QMBs, the response arises from both enantioselective and non-enantioselective interactions. At the same time, each pair of receptor enantiomers is supposed to respond similarly only to the non-enantioselective interactions.

A common technique to recognize two enantiomers of the same compound involves measuring the circular dichroism. Indeed, an active optical enantiomer absorbs slightly different clockwise and anticlockwise circularly polarized light and vice versa for the other enantiomer. The difference in absorbance due to the chirality of enantiomers is even three-four orders of magnitude lower than the total absorbance of the medium, and ad hoc measure units, such as theta, are utilized to indicate the chiral nature of compounds. The ellipticity, termed θ, is calculated as
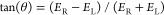
1where *E*_R_ and *E*_L_ are the magnitudes of the electric field vectors of the R- and L-circularly polarized light. Similarly, here we proposed a normalization based on the interaction of an enantiomer of a chiral analyte with the two enantiomers of a receptor rather than with the two polarizations of light. Indeed, the two enantiomers of a receptor molecule may reveal the chiral nature of an analyte if they have specular patterns of selectivity toward it. Mathematically, the proposed normalization rejects this common component of the responses in the sensor enantiomer pairs since they are likely due to non-enantioselective contribution.

Looking at the denominator of eq 1, we notice the sum of ER and EL. Here we propose, to the best of our knowledge for the first time, a normalization using as the denominator the mean of adduct enantiomer responses, (*R*_RR_ + *R*_SS_)/2, which we name as virtual racemic sensor reference. As racemic mixtures are 50:50 mixtures of both enantiomers, here we consider the racemic sensor references as a virtual sensor whose response is a 50:50 mixture of the magnitude of responses provided by the two sensor enantiomers. For each of the four metalloporphyrins, a virtual racemic sensor, , is created by averaging the response of (*R*,*R*)-cycHC[8] and (*S*,*S*)-cycHC[8] based films, *R*_R_ and *R*_S_, respectively.
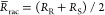
2

Normalized responses *R*_Rn_ and *R*_Sn_ can be calculated as follows
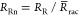
3
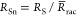
4

It is worth noting that quantification secured by QMB allows almost completely compensating for the differences in the amount of sensing material spotted for both enantiomers of each receptor, ensuring almost perfect virtual racemic sensors, which are able to compensate for the dispersion of samples due to concentration changes almost entirely. The Supporting Information reports full details and mathematical demonstration of the normalization proposed here (Paragraph 4 in the Supporting Information).

Figures 4 and 5 show two examples of the data distributions obtained with the proposed normalization, whereas all normalized data are shown in Figure S13. From the above-mentioned figures, it is possible to observe that a clear separation for limonene enantiomers occurs in all normalized sensors, whereas ZnOEP derivatives almost perfectly recognize amine enantiomers. Remarkably, the expected specularity between the enantiomeric receptors is enhanced after the normalization process since it may compensate for fluctuations from non-chiral sources, such as ancillary variation in concentrations or environmental conditions that may occur in single samples. This first outcome demonstrates the possibility of correctly estimating even minor differences in sensing responses in the films based on antipodal receptors to improve the recognition of enantiomers. Calculations of normalization effects and benefits on data coming from enantiomeric pair of sensors is reported in paragraph 4 of the Supporting Information, where theoretical and experimental outcomes have been compared.

It is worth noting that we also tested a general normalization method based on one of the two achiral sensors in the array as a reference to normalize the chiral sensor response *R̅* with respect to concentration as follows
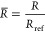
5where *R* and *R*_ref_ are the responses to a sample of chiral and achiral sensors, respectively. Indeed, the use of ZnTPP and bis-ZnOEP sensors as references improves the separation between enantiomer distributions (see Figures S14 and S15); the distributions still present a partial overlapping. The main issue with this generic normalization is the sensitivity subsisting between sensors and the reference, which only partially compensates for the concentration trend (e.g., correlations are reported in Table S4).

### 3.4 Sample Classification

**Figure 6:**
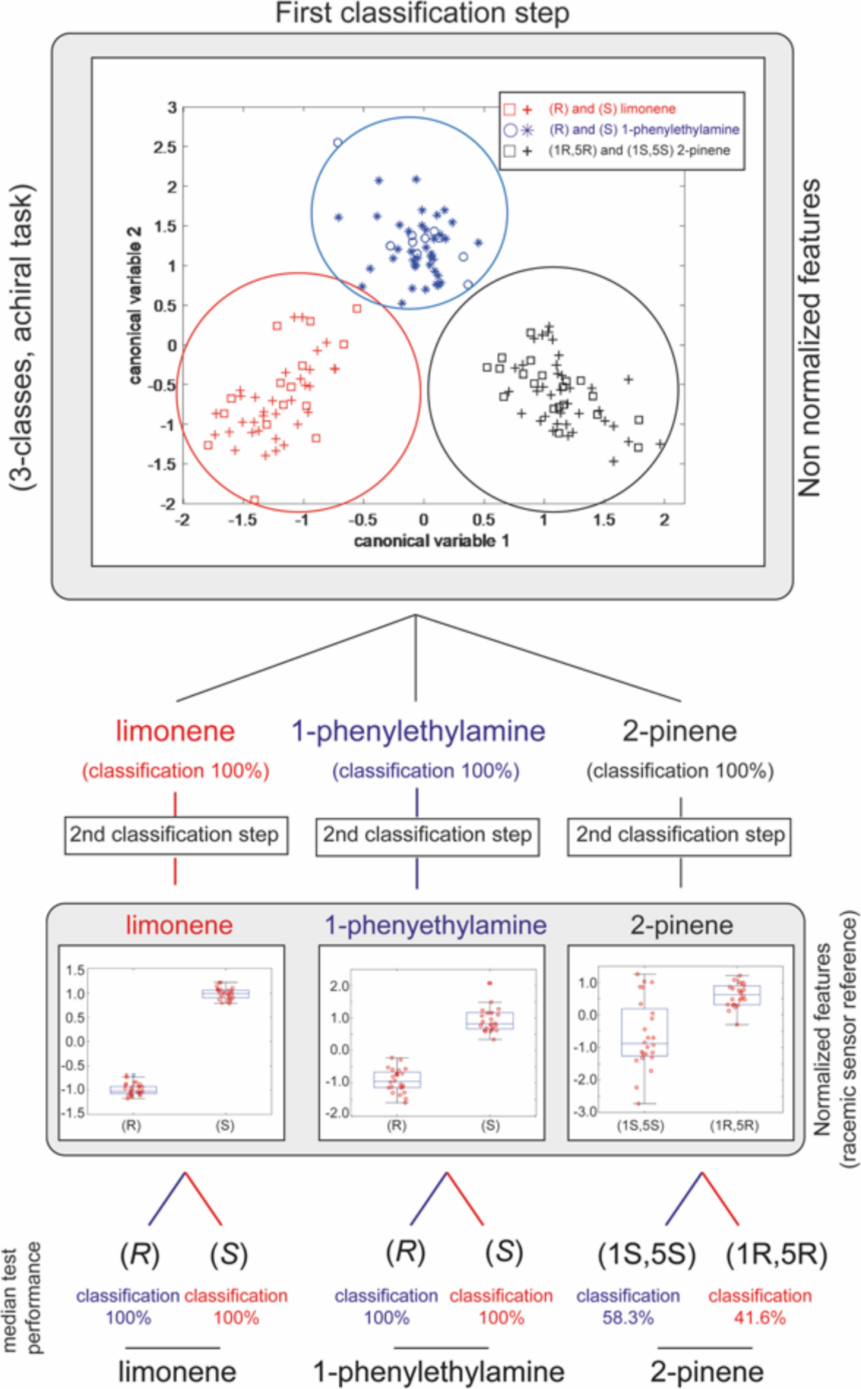
Classification protocol utilizes two steps to recognize the chemical and chiral nature of volatile compounds. The first model utilizes all features without normalization as in typical enoses. The second step utilizes only normalized features by using the virtual racemic reference introduced here. Median classification accuracies over 100 runs are reported in the figure. Overall classification results are reported in the Supporting Information. During each run, a Matlab algorithm generated new training and test datasets. Training and testing sets do not share samples with the same concentration.

To combine classic and novel multivariate gas recognition approaches, we performed classification by utilizing a two-step protocol (as reported in Figure 6). Initially, compounds were considered disregarding their chirality, obtaining a three-class task to recognize limonene, 1-phenylethylamine, and 2-pinene. Here, the classification is made by using the non-normalized features of the ten sensors. The first step plot in Figure 6 reports the projection of sensor data in the plane of the first two canonical variables obtained by utilizing non-normalized data. Notably, the three compounds (limonene, 2-pinene, and 1-phenylethylamine) are well separated. However, any separation among enantiomers does not appear evident. In other words, canonical variables of non-normalized responses well represent the distribution and separation of data without considering the chiral identity of compounds.

Once a sample is assigned to a specific class, the second model further assigns it to one of the two enantiomeric classes by using, in this case, the normalized responses of chiral sensors. Panels in Figure 6 show how the distributions of enantiomers can be separated when normalized data are utilized in the case of limonene and 1-phenylethylamine.

In Figure 6, the median classification rates of different cases and steps are reported. Performance was evaluated considering 100 randomly generated training and test subsets from the overall data. The performance of the proposed approach was tested in a challenging scenario where concentrations included in the test were not included in the training and vice versa. In this case, the concentrations appearing in the test and training dataset were randomly selected before each of the 100 runs.

Summaries of the concentrations selected and the number of samples are reported in Table S5. The linear discrimination analysis model was built on training data and applied to test ones. The accuracies for the two steps and different scenarios are reported in Figure S16. Results show that the system acts as a “classic” enose, recognizing the three classes of compounds with a perfect score in almost all cases and as an enantioselective array since it can recognize the chiral identity of limonene and 1-phenylethylamine with high accuracy, disregarding the concentrations. Notably, the normalization allows an accurate classification even if the test is considered to be non-measurable or has out-of-range concentrations with respect to the training. Indeed, in the limonene and amines cases, the model sporadically fails to predict one sample during the test phase only when training and test include completely different ranges of concentrations (see the Supporting Information for concentrations considered).

A last consideration should concern the limit of detection of an enantiomer. In principle, once a compound is correctly recognized, this approach requires that only one of the two sensors detects an enantiomer to assess its chiral nature. In the case of sensor arrays and pure known compounds, the enantiodiscrimination limit can then be estimated considering the lowest LOD for the target compound among the sensors in the array. It is worth noting that this estimation of the detection limit assumes that the sensor array correctly recognizes the target compound among the others in the model and that the enantiomers are actually separated after normalization with the virtual racemic sensor. In this context, the proposed approach potentially has LODs in the order of ten ppm for limonene and phenylethylamine enantiomers.

## 4 Conclusions

In analogy with biological systems, the results showed that using sensor arrays with the proper bivariate and multivariate analysis techniques improved the classification performances in terms of enantioselectivity, allowing the recognition of samples of limonene and 1-phenylethylamine without using highly selective sensors. The benefits are even more evident if we consider the possibility of easily obtaining films based on the receptors by mixing chiral hemicucurbituril and metalloporphyrin components. At the same time, this procedure allows tuning the chemical sensitivity by changing the porphyrin scaffold. Finally, QMB permits estimating the material cast on electrodes, validating the concept of a virtual racemic reference. These results are surely promising to extend the concept and application of electronic noses (and tongues) to enantiomer analytes, whose recognition is of great importance in medical, agrochemical, and environmental applications.

## Supporting Information Available

Additional experimental details, materials characterization, methods, pictures of experimental setup, QMB measurements, and data analysis (PDF)

## Abbreviations

ZnOEP: zinc octaethylporphyrin
ZnTPP: zinc tetraphenylporphyrin
MgTPP: magnesium tetraphenylporphyrin
cycHC[8]: cyclohexanohemicucurbit[8]uril
UV–vis: ultraviolet–visible spectroscopy
ECD: electronic circular dichroism
QMB: quartz microbalance
