# Supplementary material for: Chiral Recognition by Supramolecular Porphyrin–Hemicucurbit[8]uril-Functionalized Gravimetric Sensors
Source: ACS Appl Mater Interfaces. 2023 Jun 16;15(25):30674–83. doi: 10.1021/acsami.3c05177 (PMC10316325; doi:10.1021/acsami.3c05177)
Supplement: Supplementary file 1 — am3c05177_si_001.pdf [file am3c05177_si_001.pdf]

Supporting Information

Chiral recognition by supramolecular porphyrin - hemicucurbit[8]urils functionalized gravimetric sensors.

Gabriele Magna,[a] Marko Šakarašvili,[b] Manuela Stefanelli,[a] Gabriele Giancane,[c] Simona Bettini,[d] L. Valli,[d] Lukas Ustrnul,[b] Victor Borovkov,[b] Riina Aav,[b] Donato Monti,[e] Corrado Di Natale,[f] Roberto Paolesse[a]*

a) Department of Chemical Science and Technologies, University of Rome Tor Vergata, via della ricerca scientifica 1, 00133 Rome, Italy

b) Department of Chemistry and Biotechnology, School of Science, Tallinn University of Technology, Akadeemia tee 15, SCI-421A, Tallinn 12618, Harju maakon, Estonia

c) Department of Cultural Heritage, University of Salento, Via D. Birago, 48, I-73100 Lecce, Italy

d) Department of Biological and Environmental Sciences and Technologies, DISTEBA, University of Salento, Via per Arnesano, I-73100 Lecce, Italy

e) Department of Chemistry, Sapienza University of Rome, Piazzale Aldo Moro 5, I-00185 Rome, Italy

f) Department of Electronic Engineering University of Rome Tor Vergata, Via del Politecnico 1, 00133 Rome, Italy

Contents

1. Experimental setup S2
2. Spectroscopic characterization of Metalloporphyrins•cycHC[8] systems S3
3. QMB measurements S5
4. Normalization S14
Theoretical calculations S14
Effect of non-perfect reproducibility during sensor fabrication S17
Experimental results S19
5. Classification performances S22

List of Abbreviations

ZnOEP – zinc octaethylporphyrin
ZnTPP – zinc tetraphenylporphyrin
MgTPP – magnesium tetraphenylporphyrin
cycHC[8] – cyclohexanohemicucurbit[8]uril
UV-Vis – ultraviolet-visible spectroscopy
CD – circular dichroism
DCM – dichloromethane
QMB- Quartz Microbalance

1. Experimental setup

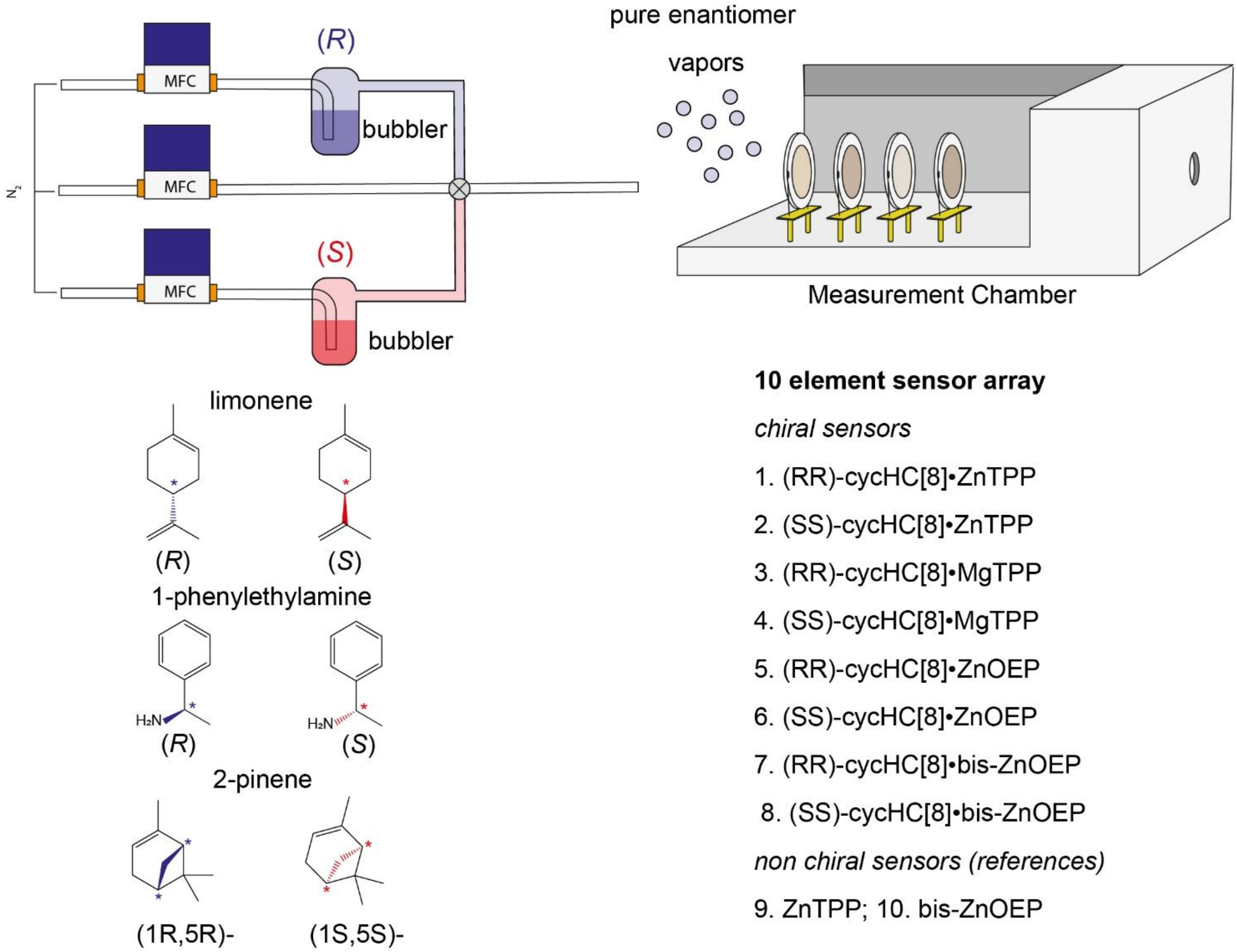

Figure S1. Sensor array setup adopted for gas sensing measurements. The array includes 8 chiral sensors (4 different pairs) and 2 non-chiral sensors.

2. Spectroscopic characterization of Metalloporphyrins•cycHC[8] systems

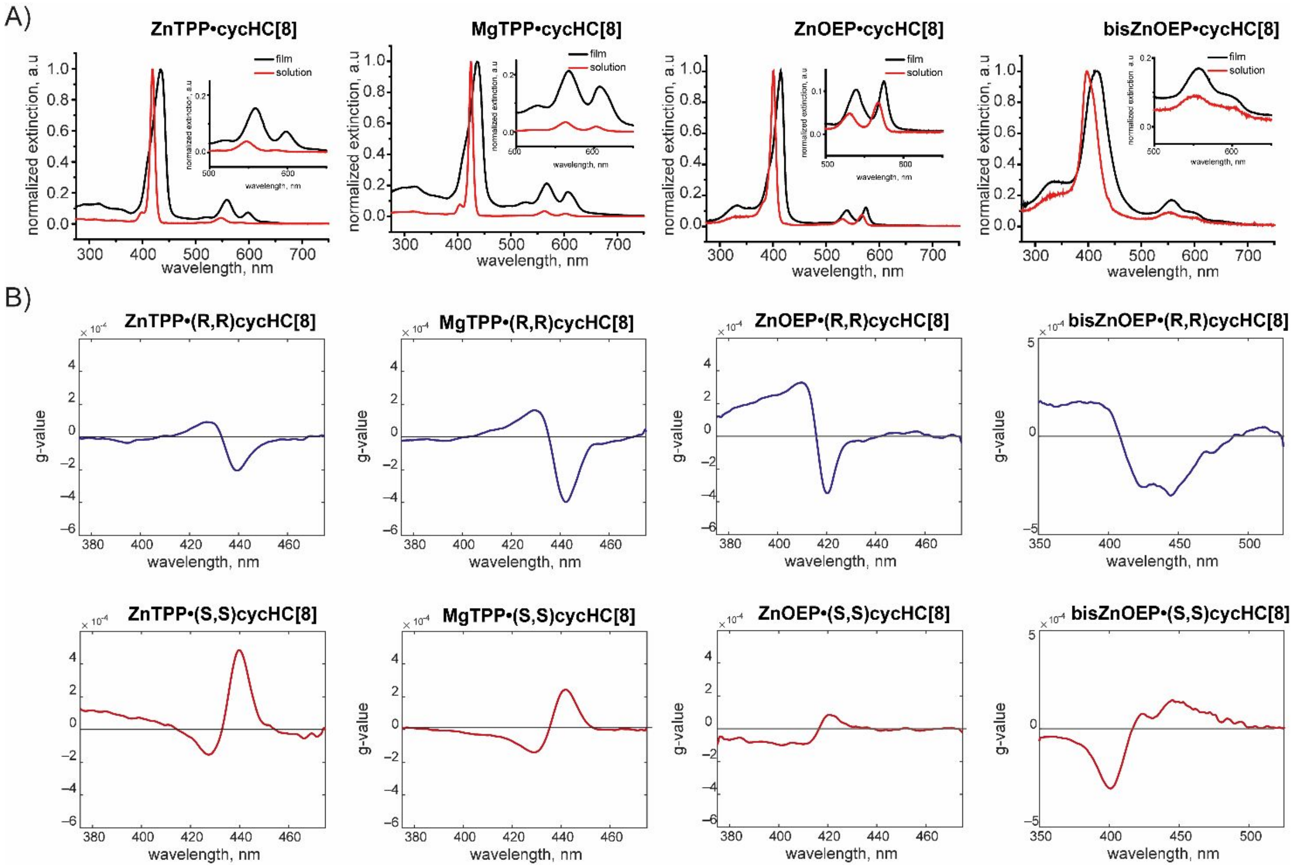

Figure S2. (A) UV-Vis spectra of metalloporphyrins•cycHC[8] systems investigated in solution and at solid state. **(B)** ECD spectra of solid films obtained by spin coating. Starting solutions in DCM did not show any detectable CD in the absorption region of porphyrins (near UV-visible).

Table S1. UV-Vis and CD bands and crossover points of the Metalloporphyrins•cycHC[8] systems investigated in solution and at solid state

		DCM solution	film on glass	
No	Metalloporphyrins•cycHC[8]	UV-Vis bands (nm)	UV-Vis bands (nm)	CD bands (nm)
1	ZnTPP	417, 547, 587	434,558, 596	427, 440 (crossover 433)
2	MgTPP	425, 563, 602	437, 567, 609	428, 441, (crossover 435)
3	ZnOEP	410, 531, 568	415, 539, 575	409, 419, (crossover 416)
4	bisZnOEP	398, 534	419, 562	446

3. QMB measurements

Table S2. List of chiral analytes and concentrations tested in QMB measurements.

Enantiomers		Concentrations [ppm]
(R)-	limonene	40, 60, 80, 100, 120, 140, 160
(S)-		
(R)-(+)-	1-phenylethylamine	76, 88, 101, 113, 126, 139
(S)-(−)-		
(1R,5S)-	2-pinene	39, 78, 117, 156, 195, 234
(1S,5S)-		

Table S3. QMB deposition details.

position	Sensing material	Initial frequency (Hz)	Final frequency (Hz)	ΔF (Hz)
QMB-1	RR-cycHC[8]@ZnTPP	20024890	19973120	51770
QMB-2	SS-cycHC[8]@ZnTPP	19989106	19942100	47006
QMB-3	RR-cycHC[8]@MgTPP	20011750	19970910	36630
QMB-4	SS-cycHC[8]@MgTPP	20014047	19975100	38947
QMB-5	RR-cycHC[8]@ZnOEP	20000572	19955100	36630
QMB-6	SS-cycHC[8]@ZnOEP	20005591	19965555	40036
QMB-7	RR-cycHC[8]@bisZnOEP	19999500	19950010	49490
QMB-8	SS-cycHC[8]@bisZnOEP	20009115	19967500	41615
QMB-9	ZnTPP	19992207	19954567	37640
QMB-10	bisZnOEP	19997583	19954005	43578

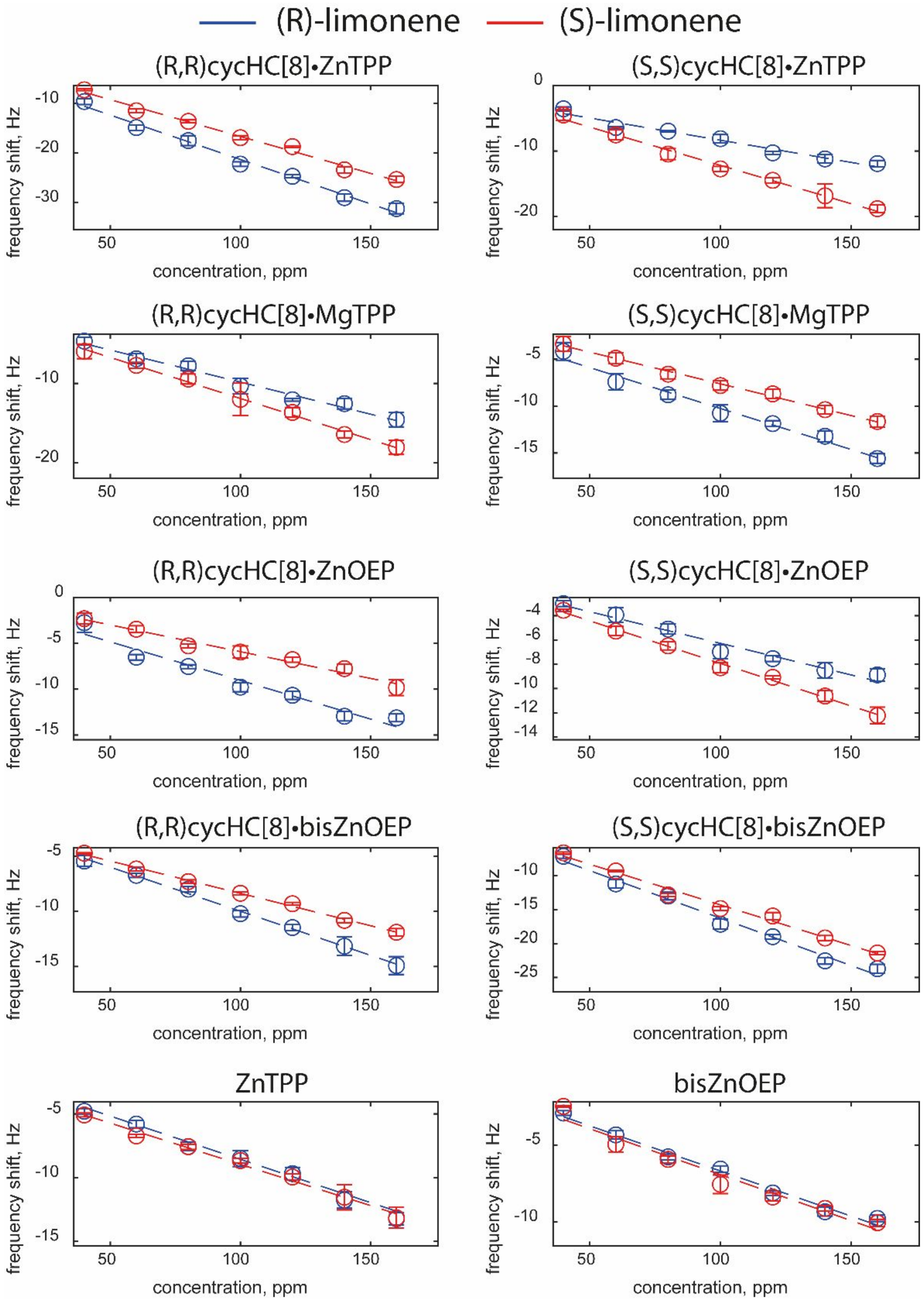

Figure S3. Characteristic curves of the ten sensors in the array to different concentrations and repetitions of limonene enantiomers' vapor. Error bars are referred to the standard error calculated over repetitions.

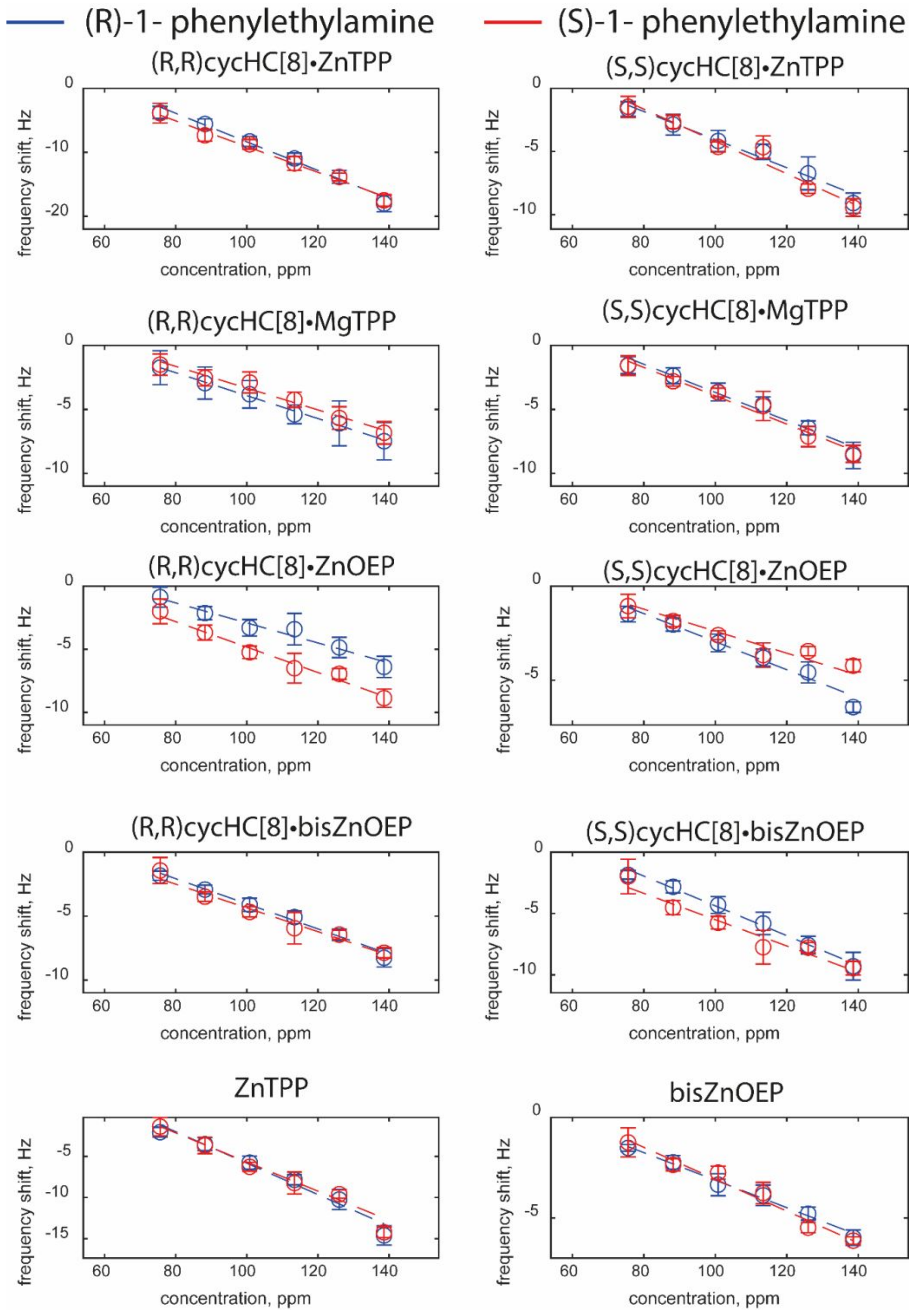

Figure S4. Characteristic curves of the ten sensors in the array to different concentrations and repetitions of 1-phenylethylamine enantiomers' vapor. Error bars are referred to the standard error calculated over repetitions

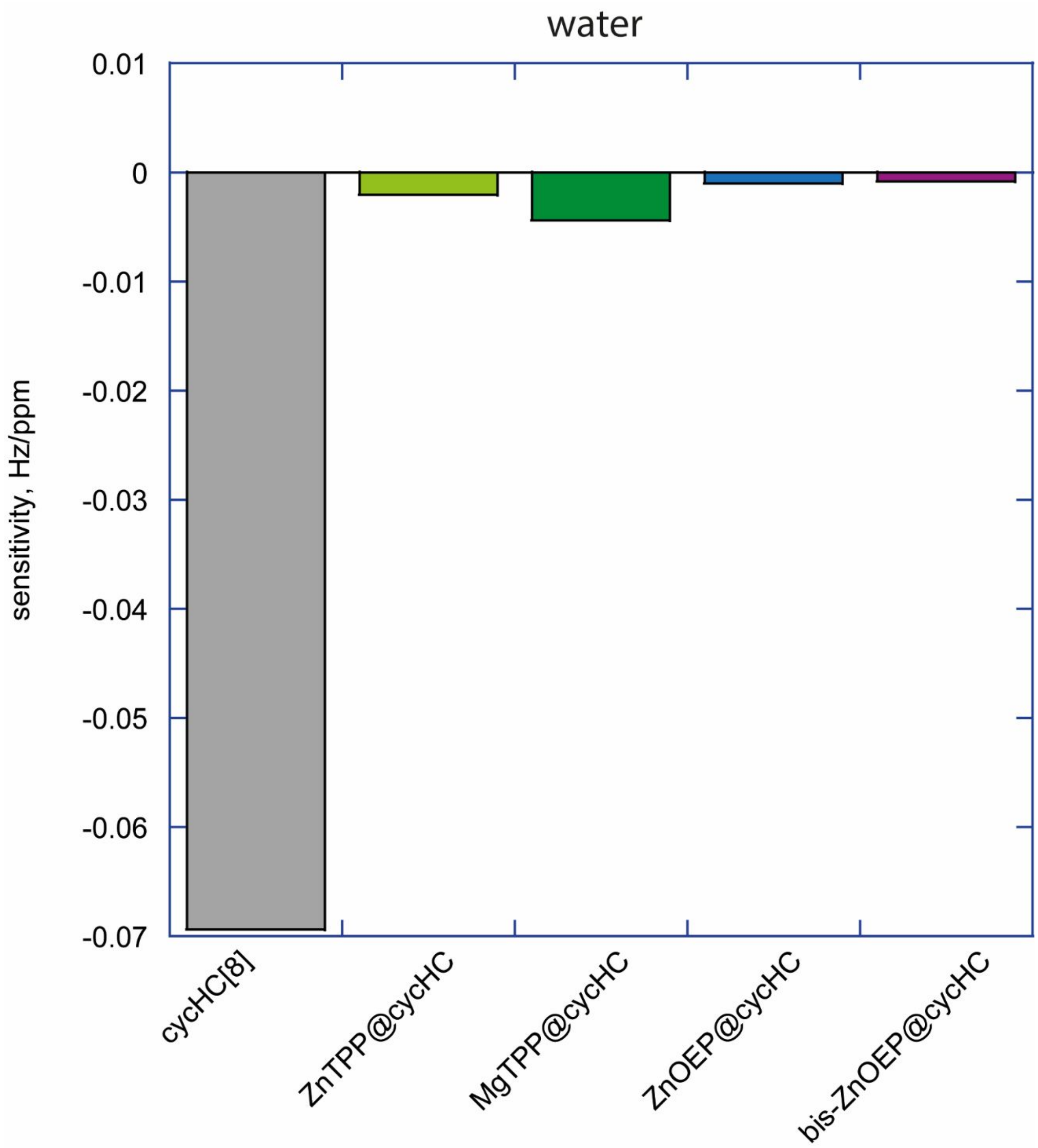

Figure S5. Bar plot reporting the sensitivity to water in the case of pure cycHC[8] films and adducts made of metalloporphyrins•cycHC. In the case of adducts, (R,R) and (S,S) enantiomers showed similar sensitivities so the mean value is reported for the sake of simplicity.

Hemicucurbituril perfomances

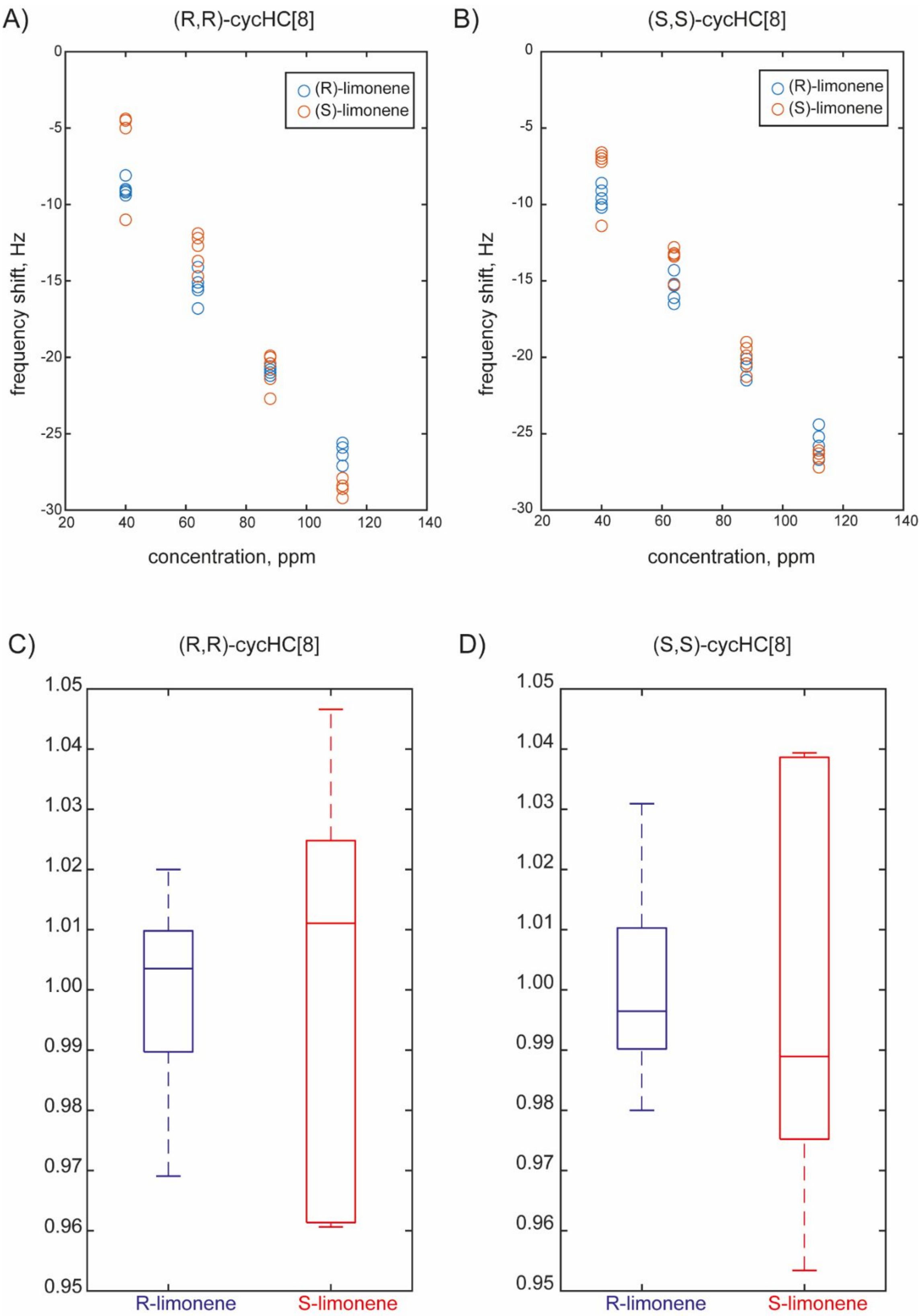

Figure S6. Response vs. concentration data of QMB sensors coated with films made with pure (R,R)- (panel A) and (S,S)-cycHC[8] (panel B) when exposed to limonene enantiomer vapors. Panels C) and D) report the distribution of enantiomer samples after the normalization using the virtual racemic references. It is possible to observe the absence of discrimination.

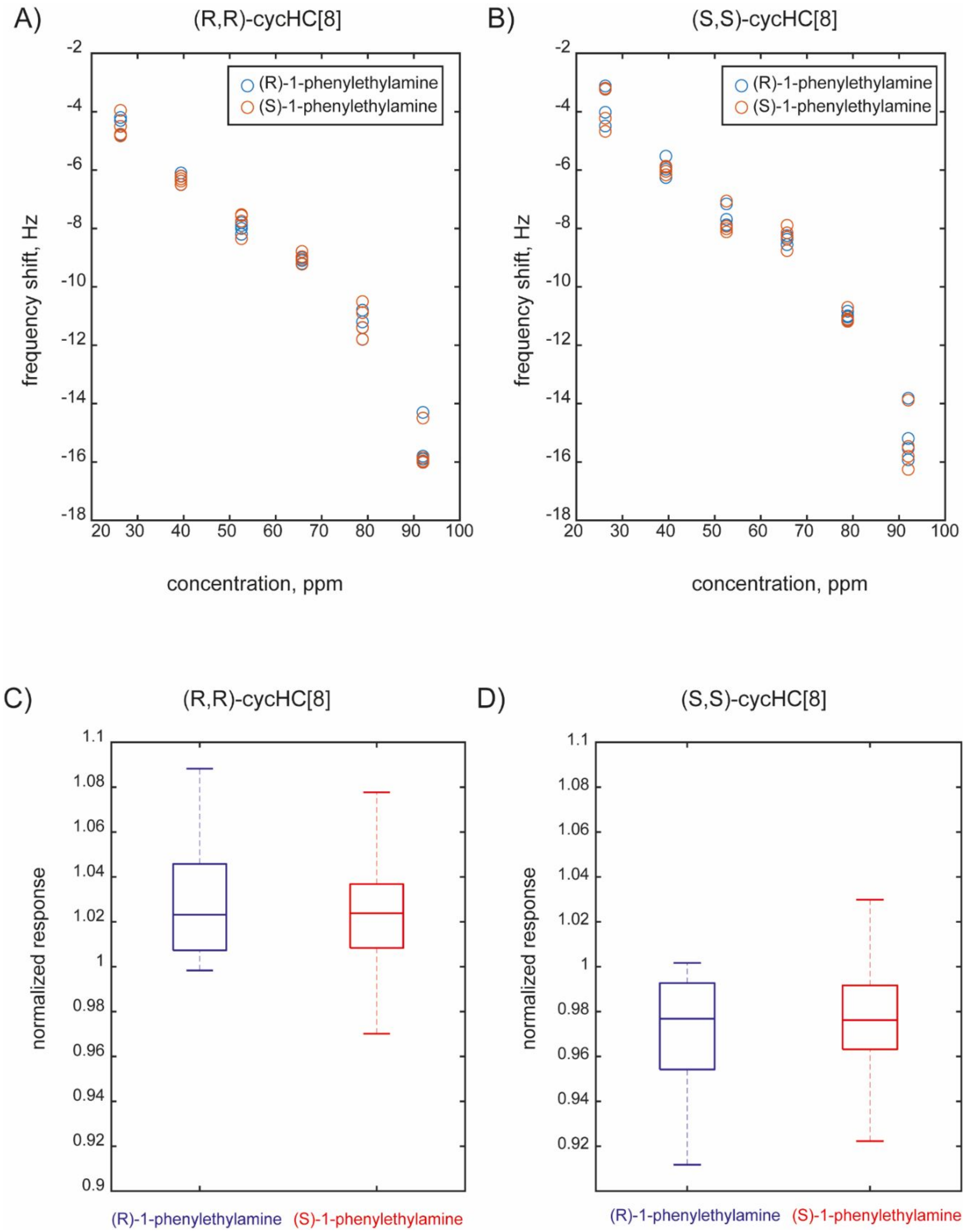

Figure S7. Response vs. concentration data of QMB sensors coated with films made with pure (R,R)- (panel A) and (S,S)-cycHC[8] (panel B) when exposed to 1-phenylethylamine enantiomer vapors. Panels C) and D) report the distribution of enantiomer samples after the normalization using the virtual racemic references. It is possible to observe the absence of discrimination.

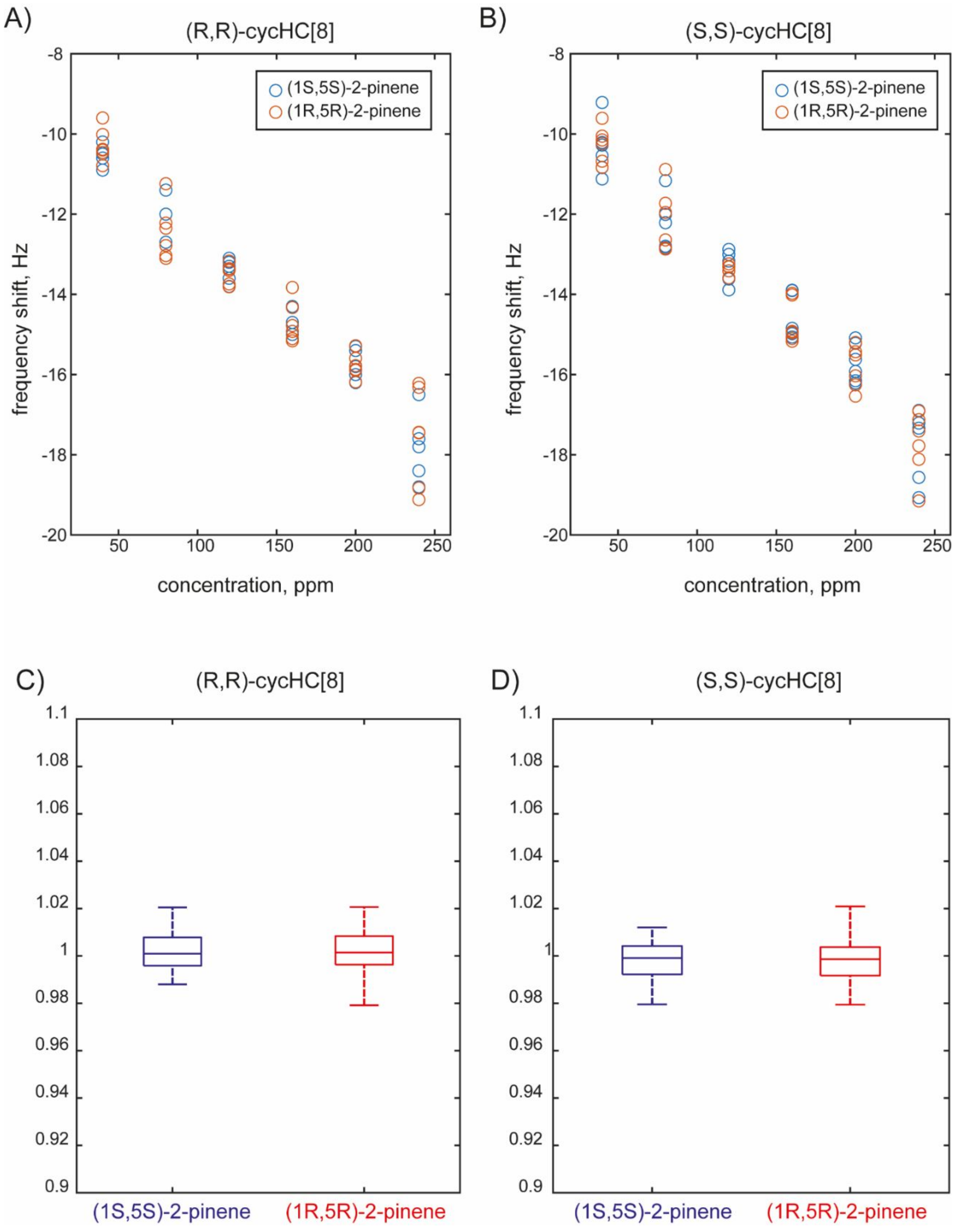

Figure S8. Response vs. concentration data of QMB sensors coated with films made with pure (R,R)- (panel A) and (S,S)-cycHC[8] (panel B) when exposed to 2-pinene enantiomer vapors. Panels C) and D) report the distribution of enantiomer samples after the normalization using the virtual racemic references. It is possible to observe the absence of discrimination.

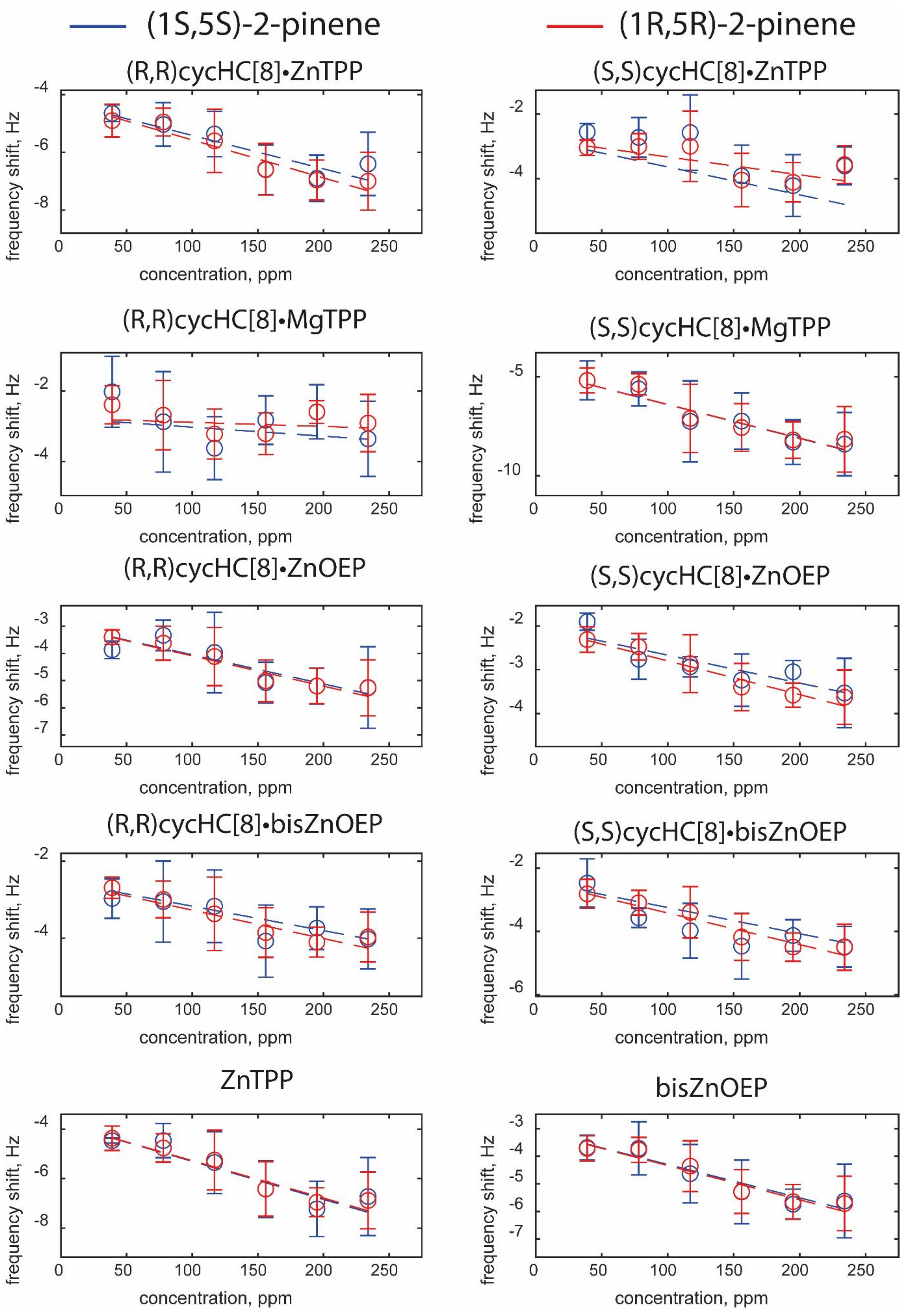

Figure S9. Characteristic curves of the ten sensors in the array to different concentrations and repetitions of 2-pinene enantiomers' vapor. Error bars are referred to the standard error calculated over repetitions.

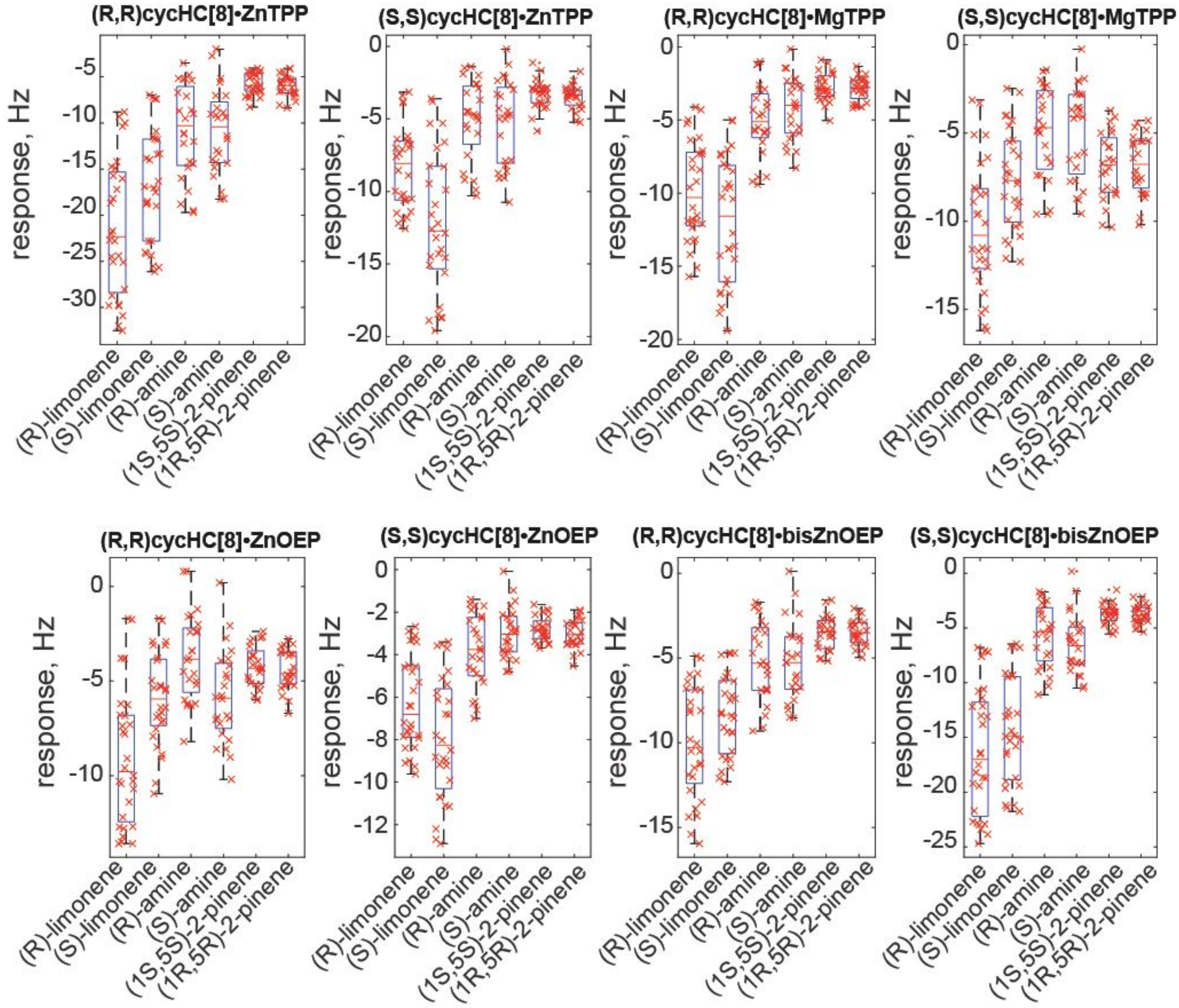

Figure S10. Data distributions of compounds tested considering each chiral sensor considered in the array. Asterisks indicate where the p-value is < 0.05, according to the Kruskal-Wallis test for the three different enantiomer pairs.

4. Normalization

Theoretical calculations

Let us consider the simple case where the response of a sensor is linearly correlated with the concentration of the enantiomer **E1**; then, we can write:

$R_1 = C_{E1} \times S_{11}$

where R_1 is the response of sensor 1, C_{E1} is the concentration of enantiomer 1, and S_{11} is the sensitivity of sensors 1 toward E_1.

In the case of a chiral receptor, we can ideally subdivide the response into two components, one due to the chiral interactions and the second produced by achiral binding events.

$R_{11} = C_{E1} \times S + C_{E1} \times S_{11}$

where $C_{E1} \times S$ addend considers the response due to non-enantioselective interactions, and $C_{E1} \times S_{11}$ is the enantioselective component of the overall sensor response.

Now, if we consider the two enantiomers of a receptor, 1 and 2, we can suppose that they will equally respond to achiral stimuli or interactions. On the other hand, they are supposed to differ by the enantioselective contribution. Thus we can write:

$R_{11} = C_{E1} \times S + C_{E1} \times S_{11}$

$R_{21} = C_{E1} \times S + C_{E1} \times S_{21}$

In this case, the two responses differ because of $C_{E1} \times S_{11}$ and $C_{E1} \times S_{21}$ terms.

Finally, if we consider the response to the second enantiomer E2, we can suppose again that the achiral component of the responses is unchanged, then the responses of two sensors can be written as:

$R_{12} = C_{E2} \times S + C_{E2} \times S_{12}$

$R_{22} = C_{E2} \times S + C_{E2} \times S_{22}$

In the case of enantiomeric sensors, we suppose that, in the presence of enantioselective interactions, one sensor responds more to one enantiomer, and the second sensor responds more to the other enantiomer of an analyte. This behavior can be easily described by considering $S_{11}>S_{21}$ and $S_{12}<S_{22}$, for example.

In this case, the characteristic curves of the two sensors will result as the ones reported in Figure S7 panel A. It is evident that a single sensor is insufficient to recognize a chiral compound nature in this situation. Indeed, if we consider a relatively extended interval of concentrations, the responses of a sensor to E1 and E2 will overlap, as graphically shown in Figure S7 panel B. The data spread is the product of sensitivity by the delta of concentrations in the interval.

Here we propose a simple normalization to utilize the responses of an enantiomeric pair of receptors to resolve the chiral identity of a compound.

Let us now consider the average response R_{M1} between the two sensor responses R_{11} and R_{21}, toward the enantiomer E1.

$$R_{rac1} = \frac{R_{11} + R_{21}}{2} = \frac{2SC_{E1} + (S_{11} + S_{21}) * C_{E1}}{2} = S * C_{E1} + \left(\frac{S_{11} + S_{21}}{2}\right) * C_{E1}$$

Finally, the proposed normalization divided the value f each sensor response by this meaning value. For example, in the case of R_{11} we obtain:

$$R_{11n} = {}^{R11}\!/_{Rrac1} = \frac{S * C_{E1} + S_{11} * C_{E1}}{S * C_{E1} + \left(\frac{S_{11} + S_{21}}{2}\right) * C_{E1}} = \frac{2 * S * C_{E1} + 2 * S_{11} * C_{E1}}{2 * S * C_{E1} + (S_{11} + S_{21}) * C_{E1}}$$

$$== \frac{2 * S * C_{E1} + 2 * S_{11} * C_{E1} + S_{21} * C_{E1} - S_{21} * C_{E1}}{2 * S * C_{E1} + (S_{11} + S_{21}) * C_{E1}} = 1 + \frac{S_{11} - S_{21}}{2 * S + (S_{11} + S_{21})}$$

If we consider all the possible combinations between the two enantiomers of receptors and analytes, we obtained the following four normalized responses:

$$R_{11n} = 1 + \frac{S_{11} - S_{21}}{2 * S + (S_{11} + S_{21})}$$

$$R_{21n} = 1 + \frac{S_{21} - S_{11}}{2 * S + (S_{11} + S_{21})}$$

$$R_{12n} = 1 + \frac{S_{12} - S_{22}}{2 * S + (S_{21} + S_{22})}$$

$$R_{22n} = 1 + \frac{S_{22} - S_{12}}{2 * S + (S_{12} + S_{22})}$$

As the first outcome, after the normalization, the responses do not depend on the concentration of analytes but only on the sensitivities of sensors. Secondly, considering the j-th enantiomer, samples are clustered only accordingly to the difference in the enantioselective interactions (S_{1j} and S_{2j}) over the sum of all sensitivity contributions (S, S_{1j} and S_{2j}) as depicted in Figure S7 panel C.

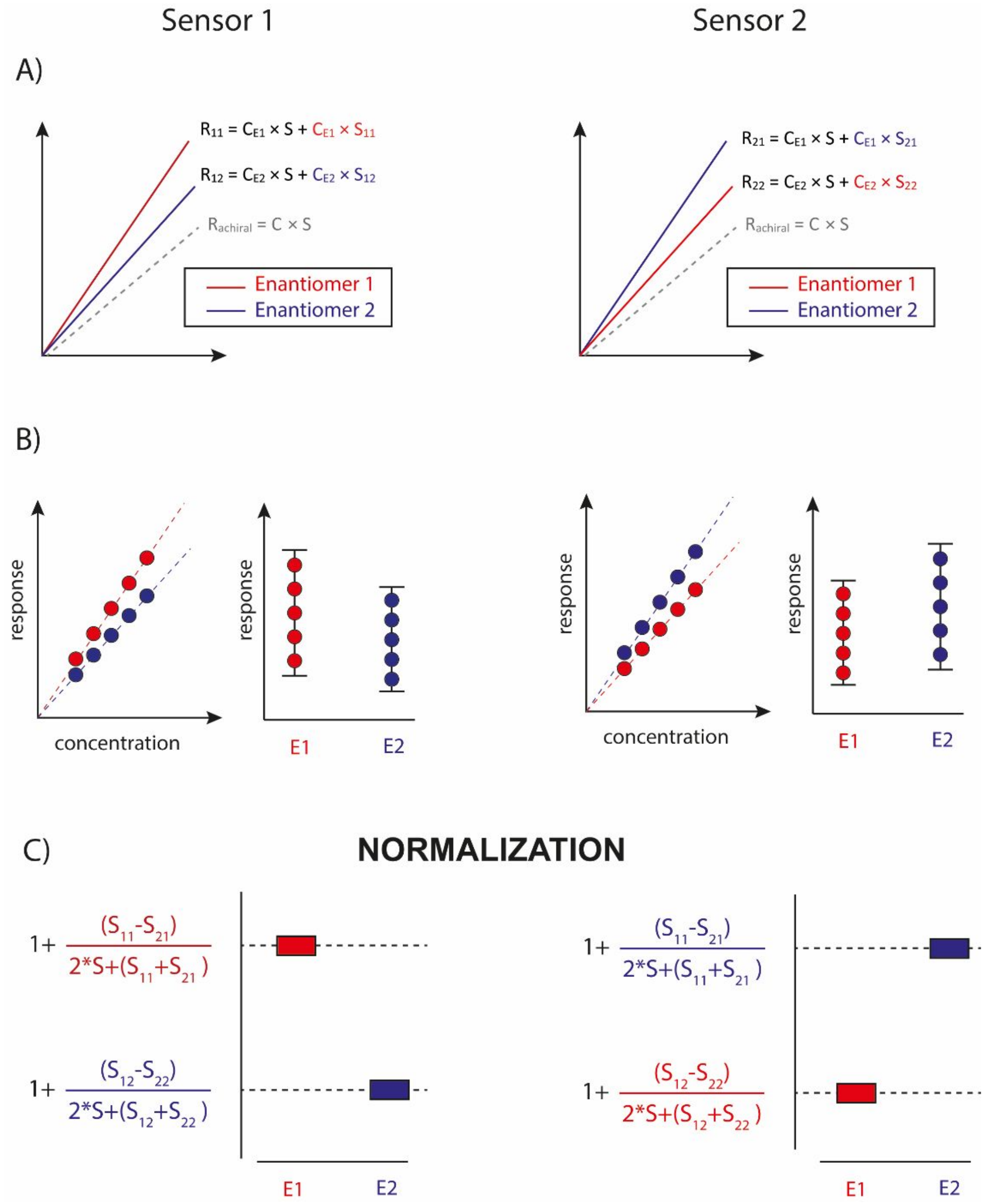

Figure S11 A) characteristic curves of low enantioselective sensors composed of two receptors with mirrored selectivity: Sensor1 has a higher affinity to enantiomer 1, and sensor 2 toward enantiomer 2. B) Effect of concentration on the distributions of sensor responses. Albeit having different mean values, the distributions of the two enantiomers cannot be separated by using a single chiral sensor. C) Effect of the proposed normalization in case the two sensors have the same non-enantioselective response component.

Effect of non-perfect reproducibility during sensor fabrication

Albeit sophisticated deposition technique, the reproducibility of sensors in terms of sensitivity is extremely difficult, if not impossible, to obtain two different sensors with the precisely same sensitivities. Thus, let us now remove the hypothesis that sensors have exactly the same sensitivity to achiral component of interactions.

$R_{11} = C_{E1} \times S_1 + C_{E1} \times S_{12}$ (response of sensor 1 to enantiomer 1)

$R_{21} = C_{E1} \times S_2 + C_{E1} \times S_{22}$ (response of sensor 2 to enantiomer 1)

$R_{12} = C_{E2} \times S_1 + C_{E2} \times S_{12}$ (response of sensor 1 to enantiomer 2)

$R_{22} = C_{E2} \times S_2 + C_{E2} \times S_{22}$ (response of sensor 2 to enantiomer 2)

Then

$$R_{rac1} = \frac{R_{11} + R_{21}}{2} = \frac{(S_1 + S_2) * C_{E1} + (S_{11} + S_{21}) * C_{E1}}{2}$$

And in the case of sensor 1 to enantiomer 1, we obtain in this case:

$$R_{11n} = {}^{R11}/_{Rrac1} = \frac{S_1 * C_{E1} + S_{11} * C_{E1}}{\frac{(S_1 + S_2) * C_{E1} + (S_{11} + S_{21}) * C_{E1}}{2}} = \frac{C_{E1} * (2 * S_1 + 2 * S_{11})}{C_{E1} * (S_1 + S_2 + S_{11} + S_{21})}$$

$$== \frac{(2 * S_1 + 2 * S_{11})}{(S_1 + S_2 + S_{11} + S_{21})} = 1 + \frac{S_1 - S_2}{(S_1 + S_2 + S_{11} + S_{21})} + \frac{S_{11} - S_{21}}{(S_1 + S_2 + S_{11} + S_{21})}$$

Repeating the passages for all sensors we obtain:

$$R_{11n} = 1 + \frac{\mathbf{S_1 - S_2}}{(\mathbf{S_1 + S_2 + S_{11} + S_{21}})} + \frac{\boldsymbol{S_{11} - S_{21}}}{(\boldsymbol{S_1 + S_2 + S_{11} + S_{21}})}$$

$$R_{21n} = 1 + \frac{\mathbf{S_2 - S_1}}{(\mathbf{S_1 + S_2 + S_{11} + S_{21}})} + \frac{\boldsymbol{S_{21} - S_{11}}}{(\boldsymbol{S_1 + S_2 + S_{11} + S_{21}})}$$

$$R_{12n} = 1 + \frac{\mathbf{S_1 - S_2}}{(\mathbf{S_1 + S_2 + S_{12} + S_{22}})} + \frac{\boldsymbol{S_{12} - S_{22}}}{(\boldsymbol{S_1 + S_2 + S_{11} + S_{21}})}$$

$$R_{22n} = 1 + \frac{\mathbf{S_2 - S_1}}{(\mathbf{S_1 + S_2 + S_{12} + S_{22}})} + \frac{\boldsymbol{S_{22} - S_{12}}}{(\boldsymbol{S_1 + S_2 + S_{12} + S_{22}})}$$

Also in this case, the two enantiomer samples are separated proportionally to the difference in enantioselective contribution over the sum of sensitivities of all sensors. The discrepancies in sensitivities toward achiral contribution results only in a shift of the responses.

In Figure S8 panel A we compare the expected separation accordingly to these simple calculations considering S1>S2. Figure S8 Panel B evidenced the experimental separation obtained after the normalization in the case of limonene and cycHC[8]•ZnTPP receptors.

In the real case, the dispersion of samples is due to the non-perfect linearities in sensor responses over a wide range of sample concentrations, noise, and reproducibility in the delivery system. However, the normalization allows the rejection of non-enantioselective interaction from the overall sensor response. At the same time, it highlights the enantioselective contribution. This result is strictly dependent on the use of both the enantiomers of a chiral recept

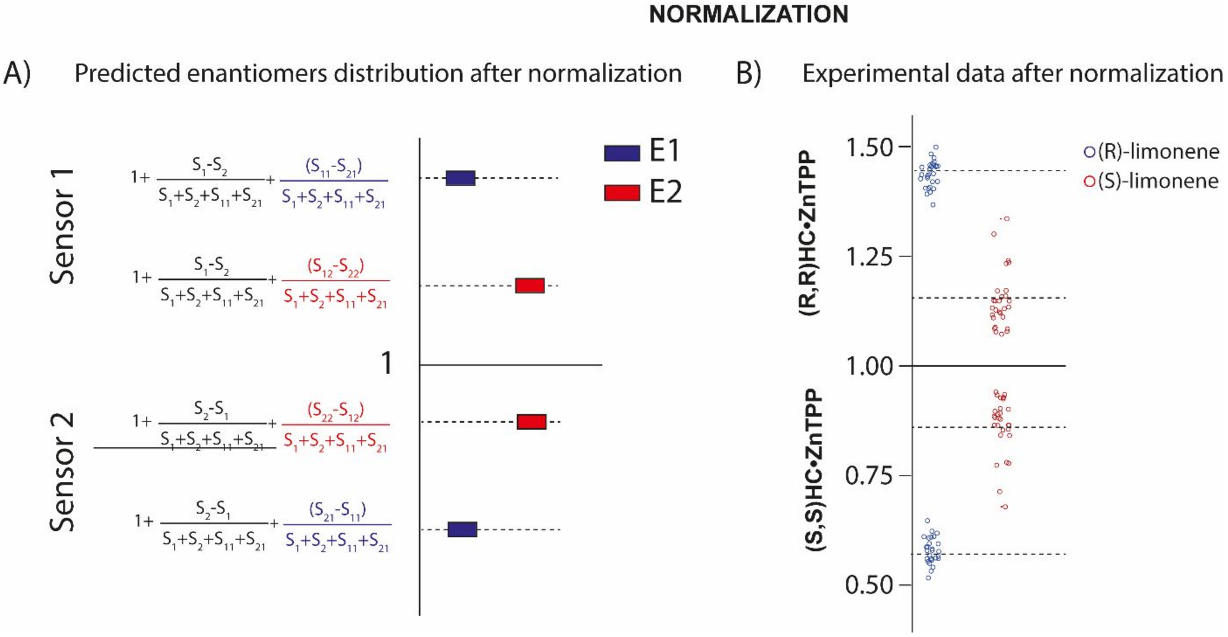

Figure S12. A) Data distribution after the normalization by virtual racemic sensors in the case of different sensitivity to non-enantioselective interactions. B) Visualization of experimental data after the normalization.

Experimental results

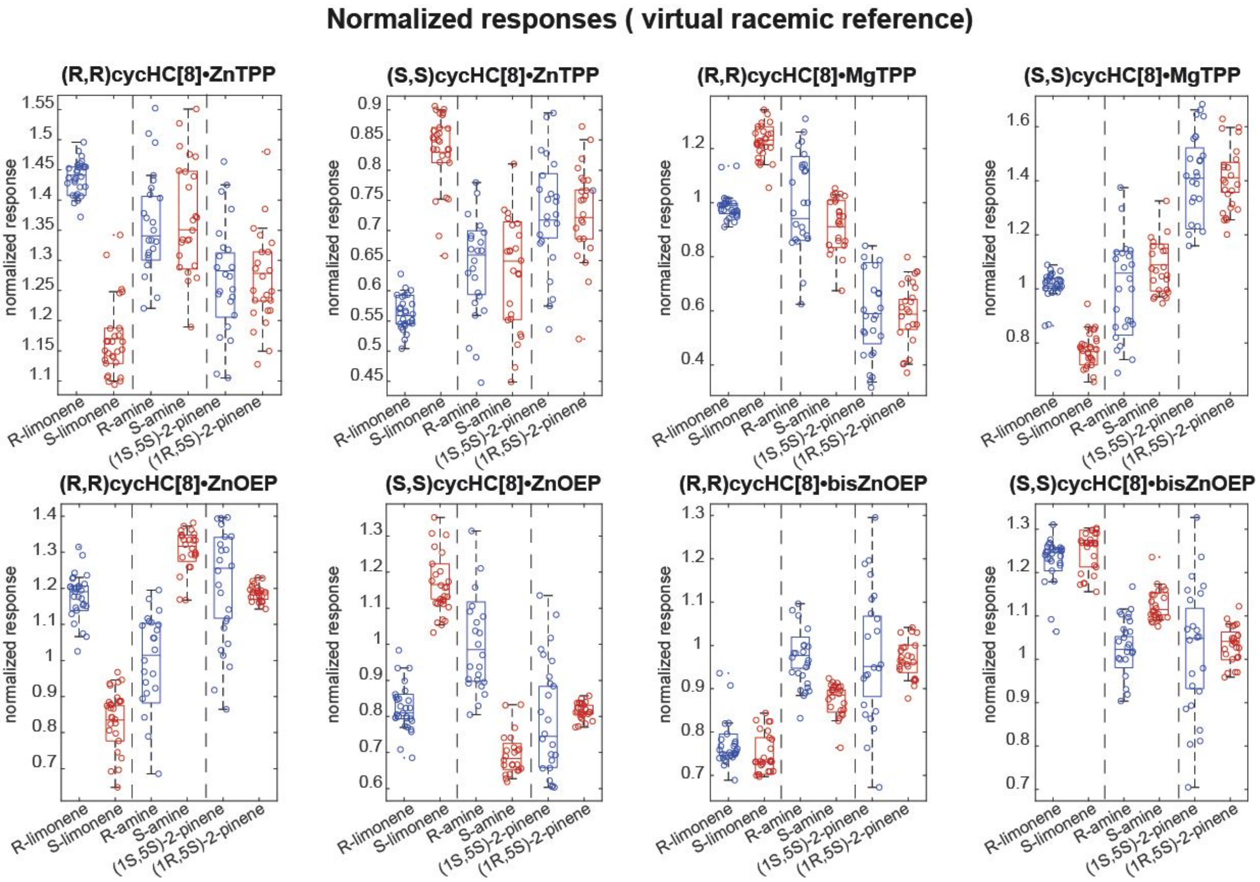

Figure S13. Data distribution after the normalization by virtual racemic sensors.

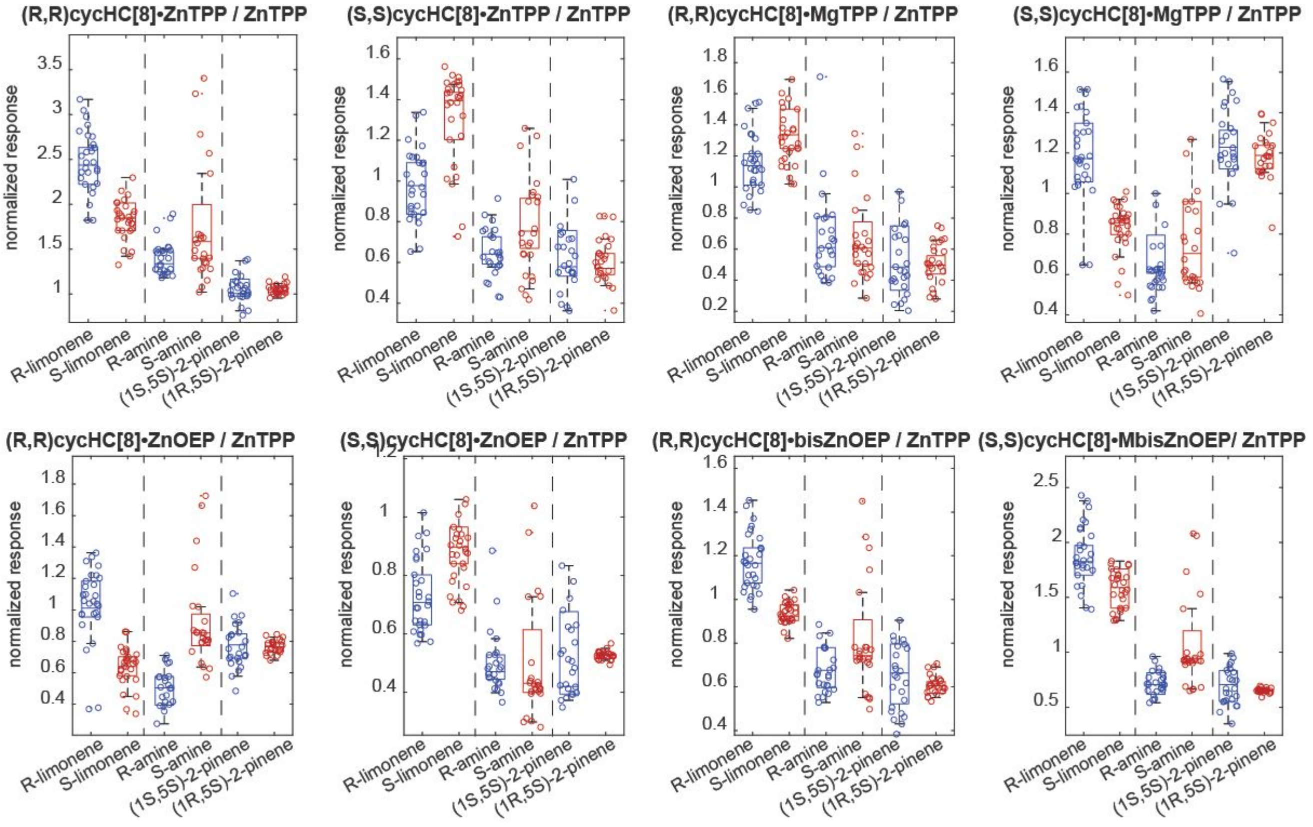

Figure S14. Data distribution after the normalization by achiral sensor based on ZnTPP.

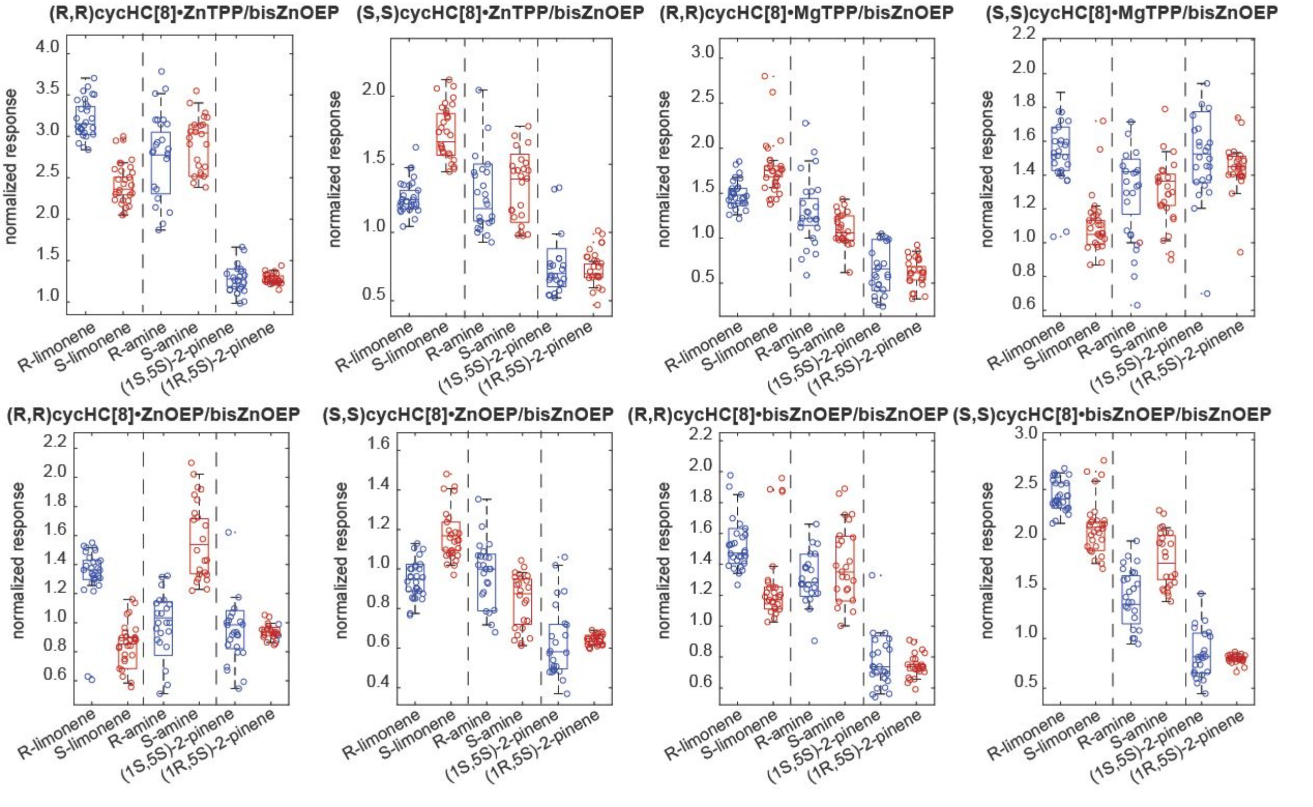

Figure S15. Data distribution after the normalization by achiral sensor based on ZnOEP

Table S4. Correlation coefficients between chiral sensors and references tested for normalization.

	Pearson correlation coefficient		
	ZnTPP	bisZnOEP	Racemic reference
RR-ZnTPP	0.8094	0.8243	0.9649
SS-ZnTPP	0.7877	0.8367	0.9626
RR-MgTPP	0.6987	0.8077	0.9150
SS-MgTPP	0.7519	0.9125	0.9210
RR-ZnOEP	0.7637	0.8371	0.9299
SS-ZnOEP	0.7678	0.8840	0.9312
RR-bisZnOEP	0.8038	0.8544	0.9929
SS-bisZnOEP	0.7177	0.8376	0.9928

Table S5. Sample subdivision into training and test datasets.

		**training**	**test**
enantiomers		concentrations [ppm]	concentrations [ppm]
(*R*)-	limonene	All the samples belonging to 4 random concentrations selected among [40, 60, 80, 100, 120, 140, 160].	The samples belonging to the 3 remaining concentrations not included in the training
(*S*)-			
(*R*)-	1-phenylethylamine	All the samples belonging to 3 random concentrations selected among [76, 88, 101, 113, 126, 139]	The samples belonging to the 3 remaining concentrations not included in the training
(*S*)-			
(1R,5R)-	2-pinene	All the samples belonging to 3 random concentrations selected among [39, 78, 117, 156, 195, 234]	The samples belonging to the 3 remaining concentrations not included in the training
(1S,5S)-			
		**Total samples = 80**	**Total samples = 72**

5. Classification performances

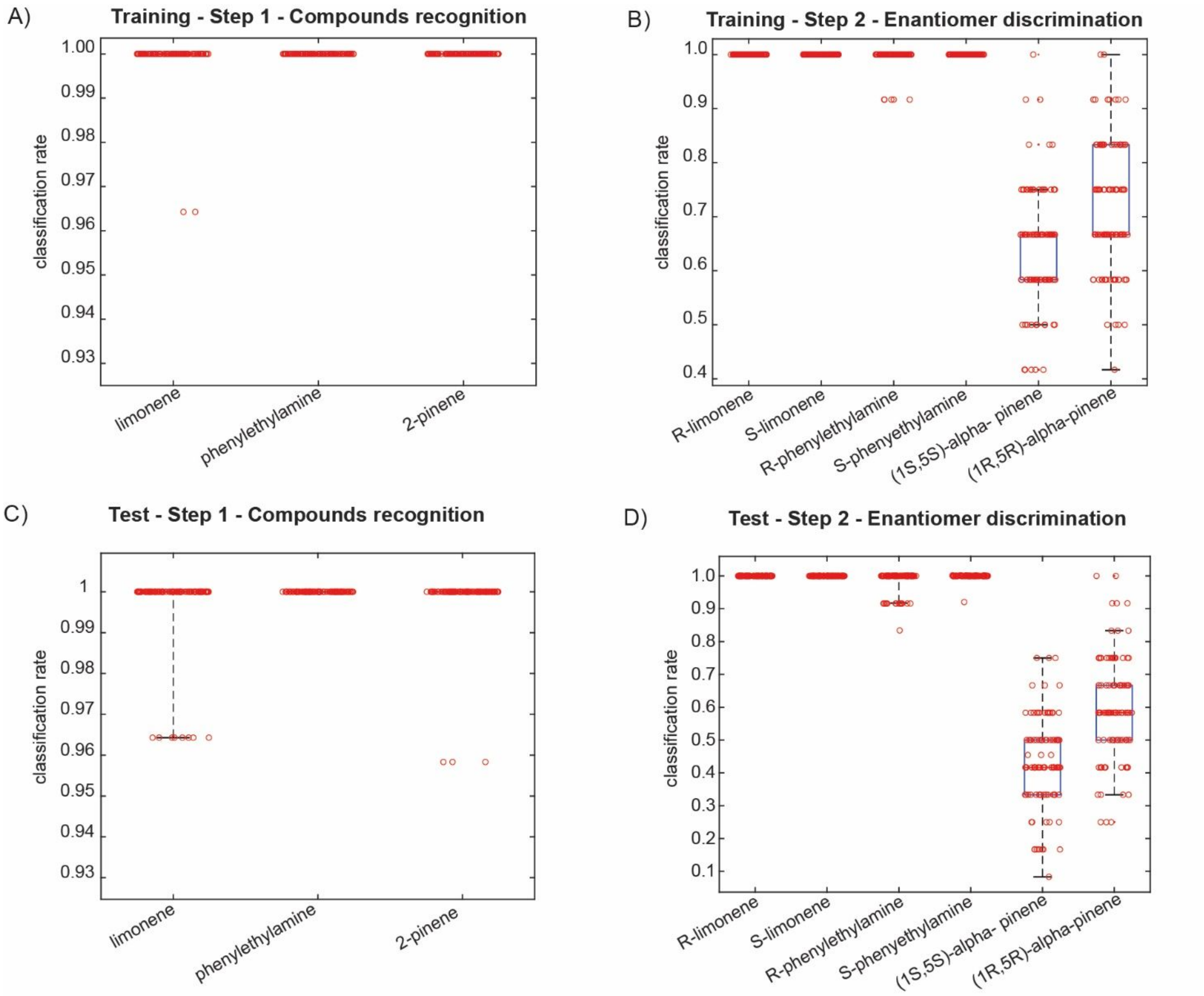

Figure S16. Classification results obtained over 100 runs where training and test dataset were data are randomly organized in training and test dataset according to instruction in Table S6.
